# Selective Anion Extraction and Recovery Using a Fe^II^_4_L_4_ Cage

**DOI:** 10.1002/anie.201800459

**Published:** 2018-02-23

**Authors:** Dawei Zhang, Tanya K. Ronson, Jesús Mosquera, Alexandre Martinez, Jonathan R. Nitschke

**Affiliations:** ^1^ Department of Chemistry University of Cambridge Lensfield Road Cambridge CB2 1EW UK; ^2^ Aix Marseille Univ CNRS Centrale Marseille, iSm2 Marseille France

**Keywords:** anion receptor, coordination cage, liquid–liquid extraction, self-assembly, supramolecular chemistry

## Abstract

Selective anion extraction is useful for the recovery and purification of valuable chemicals, and in the removal of pollutants from the environment. Here we report that Fe^II^_4_L_4_ cage **1** is able to extract an equimolar amount of ReO_4_^−^, a high‐value anion and a nonradioactive surrogate of TcO_4_^−^, from water into nitromethane. Importantly, the extraction was efficiently performed even in the presence of 10 other common anions in water, highlighting the high selectivity of **1** for ReO_4_^−^. The extracted guest could be released into water as the cage disassembled in ethyl acetate, and then **1** could be recycled by switching the solvent to acetonitrile. The versatile solubility of the cage also enabled complete extraction of ReO_4_^−^ (as the tetrabutylammonium salt) from an organic phase into water by using the sulfate salt of **1** as the extractant.

Rhenium is among the rarest elements in the Earth's crust,^1^ but it is a key ingredient for modern industry. It is used as catalyst for petroleum refining,^2^ in the high‐melting superalloys of jet engines,^3^ and in new superhard materials,^4^ to cite only three examples. The limited supply and great demand lead to a high cost, generating an economic incentive for new means to extract, separate, and recycle rhenium as perrhenate (ReO_4_^−^).^5^

Because of its similar structure and almost identical charge density, perrhenate is also used as a nonradioactive surrogate for pertechnetate (^99^TcO_4_^−^),^6^ which is an important radiopharmaceutical and one of the most problematic radioactive ions in nuclear waste.^7^ Significant advances have been made in designing sorbent materials for removing ReO_4_^−^/TcO_4_^−^ from aqueous solution by liquid–solid extraction.^7^, ^8^ These solid materials take up anionic targets from water via anion exchange. An attractive alternative to such sorbents is the use of supramolecular receptors as liquid‐phase extractants,^9^ although only a few such ReO_4_^−^/TcO_4_^−^ receptors have been reported.^10^ Compared to solid‐state anion exchange materials, supramolecular extractants functioning through molecular recognition offer the potential for better selectivity toward target anions. Their flexibility in solution may provide a better size and shape match in order to optimize specific interactions between receptors and substrates.^7^ Such receptors can thus help address the major challenge in supramolecular chemistry of anion recognition in water.^11^

Most supramolecular anion extractants have been robust covalent receptors^12^ as opposed to coordination cages.^13^ Such extractants must be stable in the presence of both water and organic solvents,^14^ properties that are easier to engineer for covalent systems. Nevertheless, compared to the synthesis of covalent cages, the preparation of self‐assembled coordination capsules usually involves less synthetic complexity. The dynamic nature of coordination bonds^15^ may also enable guest release and subsequent recycling of the extractant.^16^

We recently reported the water‐soluble sulfate salt of azaphosphatrane‐based Fe^II^_4_L_4_ tetrahedron **1** (Figure 1), which can adaptively encapsulate different anions via hydrogen bonding and electrostatic interactions in water.^17^ Herein, we develop **1** as an efficient and selective extractant, capable of extracting ReO_4_^−^ in either direction between organic and aqueous phases. We also establish a simple solvent‐switching procedure that allows **1** to be disassembled, releasing its anionic cargo and allowing it to be recycled.

**Figure 1:**
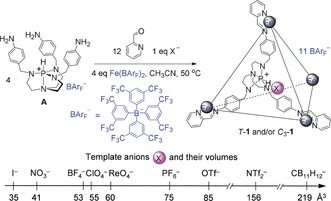
Subcomponent self‐assembly of **1** around 1 equiv of template anion.

Non‐coordinating tetrakis(3,5‐bis(trifluoromethyl)phenyl)borate (BAr_F_^−^) was selected as the counter‐anion for **1** in this work based on its lipophilicity and bulk (Figure 1). The lipophilic nature of BAr_F_^−^ renders **1** soluble in water‐immiscible organic solvents such as nitromethane. BAr_F_^−^ is larger (968 Å^3^)^18^ than the cavity volume of **1** at its most expansive (253 Å^3^; see below), precluding competition with any of the anions discussed below.

The BAr_F_^−^ salt of subcomponent **A** (Figure 1) was obtained by anion metathesis (Supporting Information section 2.1). As was observed in water,^17^ the reaction of **A** (4 equiv) with Fe(BAr_F_)_2_ (4 equiv) and 2‐formylpyridine (12 equiv) in acetonitrile failed to give the expected cage complex **1**⋅[BAr_F_]_12_, which required an internal template anion (listed in Figure 1) for its formation.

In acetonitrile, template anions with volumes below 53 Å^3^ gave rise to both a *C*_3_‐symmetric isomer (*C*_3_‐**1**, with one azaphosphatrane ^+^P‐H group oriented away from the inner cavity and the other three pointed inward) and a *T*‐symmetric isomer (*T*‐**1**, containing four inwardly‐directed ^+^P‐H groups) (Figure S1), whereas larger anionic templates, having volumes ≥55 Å^3^, resulted in the formation of *T*‐**1** exclusively (Figure S2), as was observed in water.^17^ The initially obtained mixture of isomers in the former case is kinetically metastable and gradual interconversion between cage isomers was observed. Energy barriers of conversion in CD_3_CN at 323 K were determined to be similar to the values previously obtained in water at 298 K^17^ (Figures S3–S6).

We then tested the stability of the cage, as Tf_2_N^−^⊂ **1**⋅[BAr_F_]_11_ (Tf=CF_3_SO_2_), in ethyl acetate and nitromethane, both of which are water‐immiscible organic solvents suitable for liquid–liquid extraction experiments. Circa 65 % of **1** was observed to disassemble at a concentration of 1.5 mm in EtOAc after 4 h (Figure S7), with complete disassembly occurring at more dilute concentrations. In contrast, the cage was stable without any decomposition in CD_3_NO_2_ for at least two weeks at room temperature (Figure S9). We infer that the more polar solvent nitromethane offers a greater degree of stabilization to highly cationic **1** than does less polar ethyl acetate.^19^ Nitromethane was thus chosen as the organic solvent for liquid–liquid extractions.

Interestingly, cage reassembly was observed after evaporation of EtOAc and redissolution of **1** in CD_3_CN, indicating a reversible process (Figure S8). This phenomenon provides an original means of guest release and extractant recovery, as explored further below.

Through competitive guest exchange, we were able to gauge the relative binding affinities of different anions in CD_3_NO_2_. The following hierarchy was observed: CB_11_H_12_^−^ > ReO_4_^−^ > TfO^−^ > PF_6_^−^ > ClO_4_^−^ > Tf_2_N^−^ > BF_4_^−^ > I^−^ > NO_3_^−^ (Figures S10–S17, Table S1). This ordering differs from the one observed in water: PF_6_^−^ > ReO_4_^−^ > TfO^−^ > ClO_4_^−^ > CB_11_H_12_^−^ > Tf_2_N^−^ > BF_4_^−^ > I^−^ > NO_3_^−^,^17^ especially as regards the binding affinity of CB_11_H_12_^−^. To accommodate this largest anion, the cage framework must expand; we infer that this larger conformation in water is unfavorable because it involves greater exposure of hydrophobic surface to water. In both solvents, ReO_4_^−^ binds more strongly than other common anions, indicating potential for its selective extraction.

We obtained single crystals of **1** encapsulating the two most strongly bound anions in nitromethane, CB_11_H_12_^−^ and ReO_4_^−^. X‐ray diffraction analyses^20^ (Figure 2) showed a *T*‐symmetric framework for both structures. The structures demonstrate the flexibility of the cage skeleton, allowing adaptation to guests of different sizes. Calculated cavity volumes of 157 Å^3^ and 253 Å^3^ were obtained for the ReO_4_^−^ (volume 60 Å^3^) and CB_11_H_12_^−^ (volume 219 Å^3^) complexes, respectively (Figure S18). Cavity expansion occurs through outward motion of the azaphosphatrane faces, resulting in a more open surface having pores of ca. 2.5 Å in CB_11_H_12_^−^⊂**1**, compared to ca. 1.2 Å in ReO_4_^−^⊂**1**.

**Figure 2:**
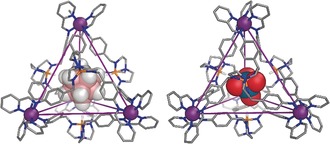
X‐ray crystal structures of CB_11_H_12_^−^⊂**1** (left) and ReO_4_^−^⊂**1** (right). Disorder, unbound counterions, non‐P‐bound hydrogen atoms, and solvents are omitted for clarity.

Since Tf_2_N^−^ is the most weakly bound among anions capable of templating *T*‐**1** exclusively, extraction of ReO_4_^−^ was initially investigated using Tf_2_N^−^⊂**1**⋅[BAr_F_]_11_ as the extractant. After mixing 0.8 mm Tf_2_N^−^⊂**1**⋅[BAr_F_]_11_ in CD_3_NO_2_ with 0.8 mm NaReO_4_ in D_2_O for 7 h, no further uptake of ReO_4_^−^ by **1** was observed. ^1^H NMR spectroscopy of the CD_3_NO_2_ phase revealed that 60 % of the ReO_4_^−^ from the aqueous phase had been extracted as ReO_4_^−^⊂**1**⋅[BAr_F_]_11_, with the remainder of **1** binding Tf_2_N^−^ (Figure S19). After displacement by the extracted ReO_4_^−^, free Tf_2_N^−^ thus transferred from CD_3_NO_2_ to D_2_O as the sodium salt.

We investigated the effect of the counterions of the Tf_2_N^−^ template by adding TBANTf_2_ (TBA=tetra‐*n*‐butylammonium), KNTf_2_, or LiNTf_2_ during the self‐assembly, but no cation effect on the efficiency of ReO_4_^−^ extraction was observed (Figure S20). Similarly, increasing the concentrations of Tf_2_N^−^⊂**1**⋅[BAr_F_]_11_ in CD_3_NO_2_ and NaReO_4_ in D_2_O to 1.3 mm (Figure S20f) did not impact extraction efficiency.

The extraction of TfO^−^ (using NaOTf) from water under identical liquid–liquid conditions was also successful but with a lower efficiency (43 %, Figure S21). Control experiments confirmed that without the cage, NaOTf did not transfer to the CD_3_NO_2_ phase (Figure S22).

In order to improve the extraction efficiency, we sought a more weakly bound template anion that avoided the complexity of generating a mixture of cage diastereomers. Such an anion was found to be *n*‐butyltrifluoroborate (^*n*^BuBF_3_^−^). We found ^*n*^BuBF_3_^−^ to be able to template *T*‐**1** exclusively (Figures S23–S28), and the resultant ^*n*^BuBF_3_^−^⊂**1**⋅[BAr_F_]_11_ to be stable in CD_3_NO_2_ for weeks. Moreover, 1 equiv of Tf_2_N^−^ in CD_3_NO_2_ almost completely displaced the encapsulated ^*n*^BuBF_3_^−^ (Figure S29), marking ^*n*^BuBF_3_^−^ as the weaker binder.

When the extractant ^*n*^BuBF_3_^−^⊂**1**⋅[BAr_F_]_11_ in CD_3_NO_2_ was mixed with an equimolar amount of NaReO_4_ in D_2_O, only ReO_4_^−^⊂**1**⋅[BAr_F_]_11_ was observed after extraction, indicating complete removal of ReO_4_^−^ from water (Figures 3 c and S30). Complete extraction of TfO^−^ from aqueous NaOTf was also achieved by using ^*n*^BuBF_3_^−^⊂**1**⋅[BAr_F_]_11_ (Figure S31).

**Figure 3:**
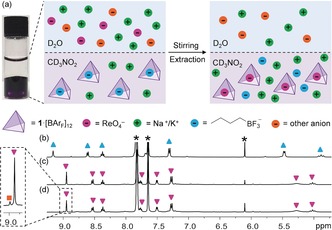
a) Selective liquid–liquid extraction of ReO_4_^−^ in the presence of other anions. Conditions: 0.8 mm ^*n*^BuBF_3_^−^⊂**1**⋅[BAr_F_]_11_ in CD_3_NO_2_; 0.8 mm in D_2_O of each of NaReO_4_, NaF, NaCl, NaBr, NaI, Na_2_SO_4_, KClO_4_, KNO_3_, NaBF_4_, NaH_2_PO_4_, and NaOAc; 7 hours stirring at RT; b–d) Partial ^1^H NMR spectra of (b) the CD_3_NO_2_ phase before extraction, showing only the presence of ^*n*^BuBF_3_^−^⊂**1**⋅[BAr_F_]_11_ (blue ▴); c) the CD_3_NO_2_ phase after extraction in the absence of competing anions, showing only the presence of ReO_4_^−^⊂**1**⋅[BAr_F_]_11_ (pink ▾); (d) the CD_3_NO_2_ phase after extraction in the presence of competing anions, showing the presence of 97 % ReO_4_^−^⊂**1**⋅[BAr_F_]_11_ (pink ▾) and 3 % ClO_4_^−^⊂**1**⋅[BAr_F_]_11_ (orange ▪). The peaks of BAr_F_^−^ and the trimethoxybenzene standard are denoted by asterisks.

Encouraged by these results, we evaluated the selectivity of **1** toward ReO_4_^−^ in the presence of 10 other different anions simultaneously in water: F^−^, Cl^−^, Br^−^, I^−^, SO_4_^2−^, ClO_4_^−^, NO_3_^−^, BF_4_^−^, H_2_PO_4_^−^, and AcO^−^ (1 equiv to ReO_4_^−^ in each case). The extraction efficiency for ReO_4_^−^ by ^*n*^BuBF_3_^−^⊂ **1**⋅[BAr_F_]_11_ in the presence of this anion library was 97 %, with ClO_4_^−^ comprising the other 3 % extracted (Figure 3).

We also developed a strategy to release and separate the extracted guest and recover the cage extractant by exploiting the instability of **1** in less polar solvents. As shown in Figure 4, after extraction, the nitromethane layer was separated and the solvent evaporated. The isolated cage was then redissolved in degassed EtOAc. As described above, the cage disassembled in this solvent. The extracted guest transferred to the water phase as KReO_4_, pairing with K^+^ from ^*n*^BuBF_3_K, allowing its removal as the phases were separated. Regeneration of ^*n*^BuBF_3_^−^⊂**1**⋅[BAr_F_]_11_, which could be reused for further extraction experiments, was realized by evaporating the ethyl acetate and adding acetonitrile, along with ^*n*^BuBF_3_K (Figure S32).

**Figure 4:**
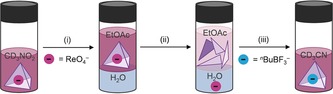
Illustration of cage extractant recycling: (i) After evaporation of CD_3_NO_2_, ReO_4_^−^⊂**1**⋅[BAr_F_]_11_ was redissolved in degassed EtOAc; degassed H_2_O was then added. (ii) After stirring for 4 h, the cage disassembled and ReO_4_^−^ was released, transferring to the H_2_O phase. (iii) After separation and evaporation of the EtOAc layer, addition of CD_3_CN and ^*n*^BuBF_3_^−^ resulted in regeneration of the extractant ^*n*^BuBF_3_^−^⊂**1**⋅[BAr_F_]_11_.

Interestingly, due to the versatile solubility of **1**, either ReO_4_^−^ or TfO^−^ could also be extracted from an organic phase into water, in the opposite direction to what was described above. In this case, Tf_2_N^−^⊂**1**⋅[SO_4_]_5.5_ as extractant completely removed either ReO_4_^−^ or TfO^−^ from CD_3_NO_2_ into D_2_O (Figures 5 and S33). Control experiments showed that without the cage, TBAReO_4_ and TBAOTf did not transfer to D_2_O (Figures S34).

**Figure 5:**
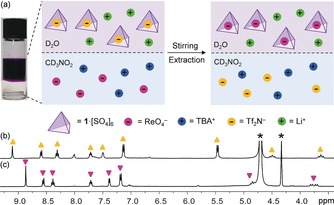
a) Illustration of the liquid–liquid extraction of ReO_4_^−^ from an organic phase into water. Conditions: 0.8 mm Tf_2_N^−^⊂**1**⋅[SO_4_]_5.5_ in D_2_O; 0.8 mm TBAReO_4_ in CD_3_NO_2_; 3 hours stirring. b,c) Partial ^1^H NMR spectra of (b) the D_2_O phase before extraction, showing only Tf_2_N^−^⊂**1**⋅[SO_4_]_5.5_ (yellow ▴), and (c) the D_2_O phase after extraction, showing only ReO_4_^−^⊂**1**⋅[SO_4_]_5.5_ (pink ▾). HDO and CHD_2_NO_2_ peaks are represented by asterisks.

In summary, we have demonstrated for the first time the feasibility of using a coordination cage for biphasic extraction. By employing BAr_F_^−^ as counter‐anion and ^*n*^BuBF_3_^−^ as template, ^*n*^BuBF_3_^−^⊂**1**⋅[BAr_F_]_11_ was capable of completely extracting ReO_4_^−^ from water into nitromethane. An efficiency of 97 % was achieved even in the presence of 10 competing anions. A novel strategy for extractant regeneration was developed by taking advantage of the differential stability of **1** across solvents. Moreover, due to the versatile solubility of **1** when paired with different counter‐anions, complete extraction of ReO_4_^−^ (TBAReO_4_) from an organic phase into water could also be accomplished by using Tf_2_N^−^⊂**1**⋅[SO_4_]_5.5_. The selective extraction properties of the cage toward perrhenate suggest great potential for recycling rhenium compounds, purification of chemicals, and for pertechnetate removal from water. Concepts developed in this study may also be generalized to enable the purification of other species using different coordination cages.

## Conflict of interest

The authors declare no conflict of interest.

## Supporting information

As a service to our authors and readers, this journal provides supporting information supplied by the authors. Such materials are peer reviewed and may be re‐organized for online delivery, but are not copy‐edited or typeset. Technical support issues arising from supporting information (other than missing files) should be addressed to the authors.

Supplementary
