# Supplementary material for: Selective Anion Extraction and Recovery Using a FeII 4L4 Cage
Source: Angew Chem Int Ed Engl. 2018 Feb 23;57(14):3717–21. doi: 10.1002/anie.201800459 (PMC6001518; doi:10.1002/anie.201800459)
Supplement: Supplementary file 1 — Supplementary [file ANIE-57-3717-s001.pdf]

Angewandte Chemie
Eine Zeitschrift der Gesellschaft Deutscher Chemiker

Supporting Information

Selective Anion Extraction and Recovery Using a $Fe^{II}_4L_4$ Cage

*Dawei Zhang, Tanya K. Ronson, Jesús Mosquera, Alexandre Martinez, and Jonathan R. Nitschke**

anie_201800459_sm_miscellaneous_information.pdf

Table of Contents

1. Materials and instrumentation 2
2. Synthesis and characterization 2
2.1 Synthesis of subcomponent **A**·BAr_F 2
2.2 Preparation of X^-⊂**1**·$[BAr_F]_{11}$ in acetonitrile 3
2.3 Kinetic study on cage isomeric interconversion 4
2.3.1 Equation derivation for free energy of activation $\Delta G^‡$ 5
2.3.2 Activation energy for NO_3^-⊂*T*-**1** ⇌ NO_3^-⊂*C*$_3$-**1** 6
2.3.3 Activation energy for BF_4^-⊂*T*-**1** ⇌ BF_4^-⊂*C*$_3$-**1** 7
2.3.4 Activation energy for I^-⊂*T*-**1** ⇌ I^-⊂*C*$_3$-**1** 7
2.4 Cage stability in EtOAc and CD_3NO_2 8
3. Competitive binding studies 9
4. X-ray crystallography 13
5. Volume calculations 16
6. Anion extraction experiments 17
6.1 Anion extraction by Tf_2N^-⊂**1**·$[BAr_F]_{11}$ 17
6.2 Anion extraction by $^nBuBF_3^-$⊂**1**·$[BAr_F]_{11}$ 20
6.2.1 Cage formation driven by the $^nBuBF_3^-$ template 20
6.2.2 Binding affinity of $^nBuBF_3^-$ relative to Tf_2N^- 23
6.2.3 Anion extraction by $^nBuBF_3^-$⊂**1**·$[BAr_F]_{11}$ 23
6.3 Cage extractant recycle 26
6.4 Anion extraction from an organic phase to water using Tf_2N^- ⊂ **1**·$[SO_4]_{5.5}$ 27
7. References 28

1. Materials and instrumentation

Unless otherwise specified, all reagents were purchased from commercial sources and used as received. $Fe(BAr_F)_2 \cdot 6CH_3CN$ was prepared as reported.[1] NMR spectra were recorded using a Bruker 400 MHz Avance III HD Smart Probe (routine ^{1}H NMR), DCH 500 MHz dual cryoprobe (high-resolution ^{13}C), and DPX S5 500 MHz BB ATM (^{1}H, ^{13}C, ^{19}F and ^{31}P NMR and 2D experiments). Chemical shifts for ^{1}H, ^{13}C, ^{19}F and ^{31}P NMR are reported in ppm on the δ scale; ^{1}H and ^{13}C were referenced to the residual solvent peak. Coupling constants (J) are reported in Hz. DOSY experiments were performed on a Bruker DPX S5 500 MHz BB ATM spectrometer. Maximum gradient strength was 6.57 G/cmA. The standard Bruker pulse program, ledbpgp2s, employing a stimulated echo and longitudinal eddy-current delay (LED) using bipolar gradient pulses for diffusion was utilized. Rectangular gradients were used with a total duration of 1.5 ms. Gradient recovery delays were 1200 µs. Individual rows of the S4 quasi-2D diffusion databases were phased and baseline corrected.

Flash column chromatography was performed using Silica Gel high purity grade (pore size 60 Å, 230-400 mesh particle size, Sigma-Aldrich). TLC analyses were performed on Merck TLC Silica Gel 60 F254 Glass plates. Product spots were visualized under UV light (λ_{max} = 254 nm). All reactions were stirred with magnetic followers. Low resolution electrospray ionization mass spectrometry was undertaken on a Micromass Quattro LC mass spectrometer (cone voltage 10-30 eV; desolvation temp. 313 K; S4 ionization temp. 313 K) infused from a Harvard syringe pump at a rate of 10 µL/min. High-resolution mass spectra were acquired using a Thermofisher LTQ Orbitrap XL.

2. Synthesis and characterization

2.1 Synthesis of subcomponent A·BAr$_F$

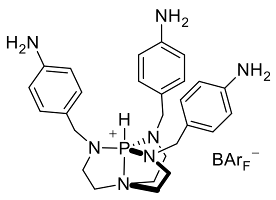

Compound **A**·Cl in its pronated form was synthesized according to previously described procedures.[2] It (150 mg) was dissolved in 5% aqueous NaOH (25 mL), followed by the addition of an excess of $NaBAr_F$ in EtOH (around 5 equiv). After stirring the reaction solution for 4 hours, the resulting mixture was extracted with dichloromethane three times. The organic phases were combined, dried over anhydrous Na_2SO_4, filtered and evaporated. After purification by column chromatography over silica gel (first with CH_2Cl_2 as eluent, then with CH_2Cl_2/CH_3OH = 90/10), **A**·BAr$_F$ was obtained as a light yellow solid with a yield > 80%. **^{1}H NMR** (CD_3CN, 298K, 500.1 MHz): δ 7.72 (s, 8H); 7.70 (s, 4H); 7.02 (d, J = 8.2 Hz, 6H); 6.65 (d, J = 8.3 Hz, 6H); 5.91 (d, J = 503.1 Hz, 1H); 4.17 (s, 6H); 4.09 (d, J = 16.9 Hz, 6H); 3.09 (q, J = 5.7 Hz, 6H); 2.97 (dt, J = 10.3, 6.2 Hz, 6H) ppm. **^{13}C NMR** (CD_3CN, 298K, 125.7 MHz): 161.6, 147.5, 134.7, 129.0, 128.8, 126.4, 124.5, 114.5, 50.5, 46.7, 38.5 ppm. **^{31}P NMR** (CD_3CN, 298K, 202.4 MHz): δ -11.2 ppm. **^{19}F NMR** (470.4 MHz, 298 K, CD_3CN): δ -63.5 ppm. **ESI-HRMS** m/z: calcd for $C_{27}H_{31}N_7O_6P$ $[M]^+$ 490.2843, found 490.2845; calcd for $C_{32}H_{12}BF_{24}$ (A^-) 863.0660, found 863.0687.

2.2 Preparation of X⁻⊂1·[BArF]11 in acetonitrile

A·BAr_F (5.4 mg, 4.0 μmol, 4 equiv), $Fe(BAr_F)_2·6CH_3CN$ (8.1 mg, 4.0 μmol, 4 equiv) and 2-formylpyridine (1.3 mg, 12 μmol, 12 equiv) were dissolved in CD_3CN (500 μL). Then 1 equiv of template anion (4.0 μmol) as the salt $TBABF_4$, $TBANO_3$, TBAI, $TBAClO_4$, $TBAReO_4$, $TBAPF_6$, TBAOTf, $TBANTf_2$/$KNTf_2$/$LiNTf_2$ or $CsCB_{11}H_{12}$, was added. The reaction mixture was kept at 323 K under nitrogen, giving rise to a purple solution of X^-⊂**1**·$[BAr_F]_{11}$. The cages used for extraction and guest displacement experiments were prepared at a 10 times larger scale.

It was found that the reaction did not give complete cage formation at room temperature. Similar to the previous results in D_2O,[2] no cage formation was observed in the absence of additional template anions at 323 K in CD_3CN. When using template anions with volumes ≤ 53 Å^3, such as BF_4^-, NO_3^- or I^-, both X^-⊂*T*-**1**·$[BAr_F]_{11}$ and X^-⊂*C*$_3$-**1**·$[BAr_F]_{11}$ were observed in solution (Figure S1). When using template anions with volumes ≥ 55 Å^3, such as ClO_4^-, ReO_4^-, PF_6^-, TfO^-, Tf_2N^- or $CB_{11}H_{12}^-$, X^-⊂*T*-**1**·$[BAr_F]_{11}$ was formed exclusively (Figure S2).

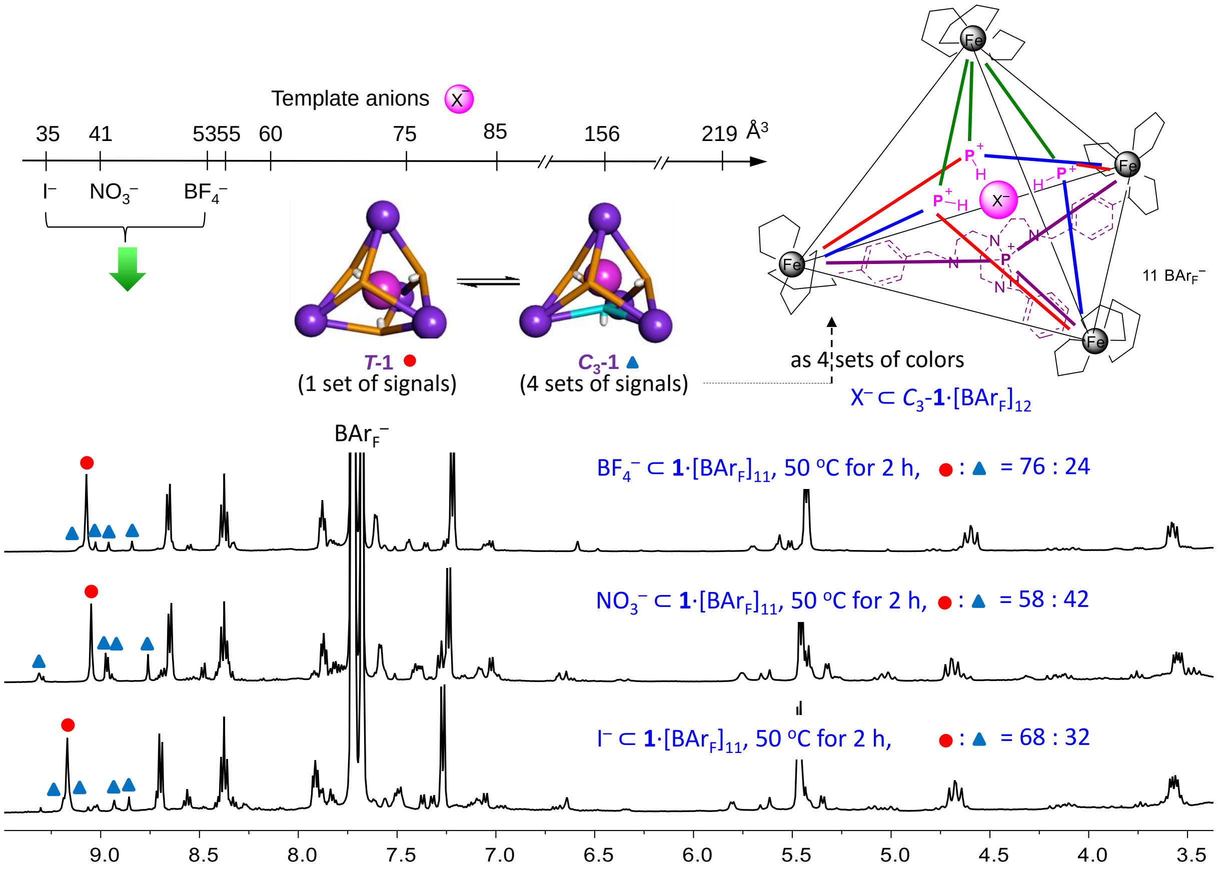

Figure S1. ^{1}H NMR (CD_3CN, 500 MHz, 298 K) spectra of the assembly of X^-⊂**1**·$[BAr_F]_{11}$ after 2 h at 298 K (X^- = I^-, NO_3^-, or BF_4^-). Two cage isomers X^-⊂*T*-**1**·$[BAr_F]_{11}$ and X^-⊂*C*$_3$-**1**·$[BAr_F]_{11}$ were observed in solution. Imine protons have been labelled in the spectra. The ratio between the two isomers in each spectrum was used as the starting point for determination of the activation energy barrier for isomeric interconversion (see Section 2.3.1). The schematic inset with four sets of colors on the four faces of the tetrahedron illustrates the origin of the four sets of signals in the ^{1}H NMR spectra of X^-⊂*C*$_3$-**1**·$[BAr_F]_{11}$.

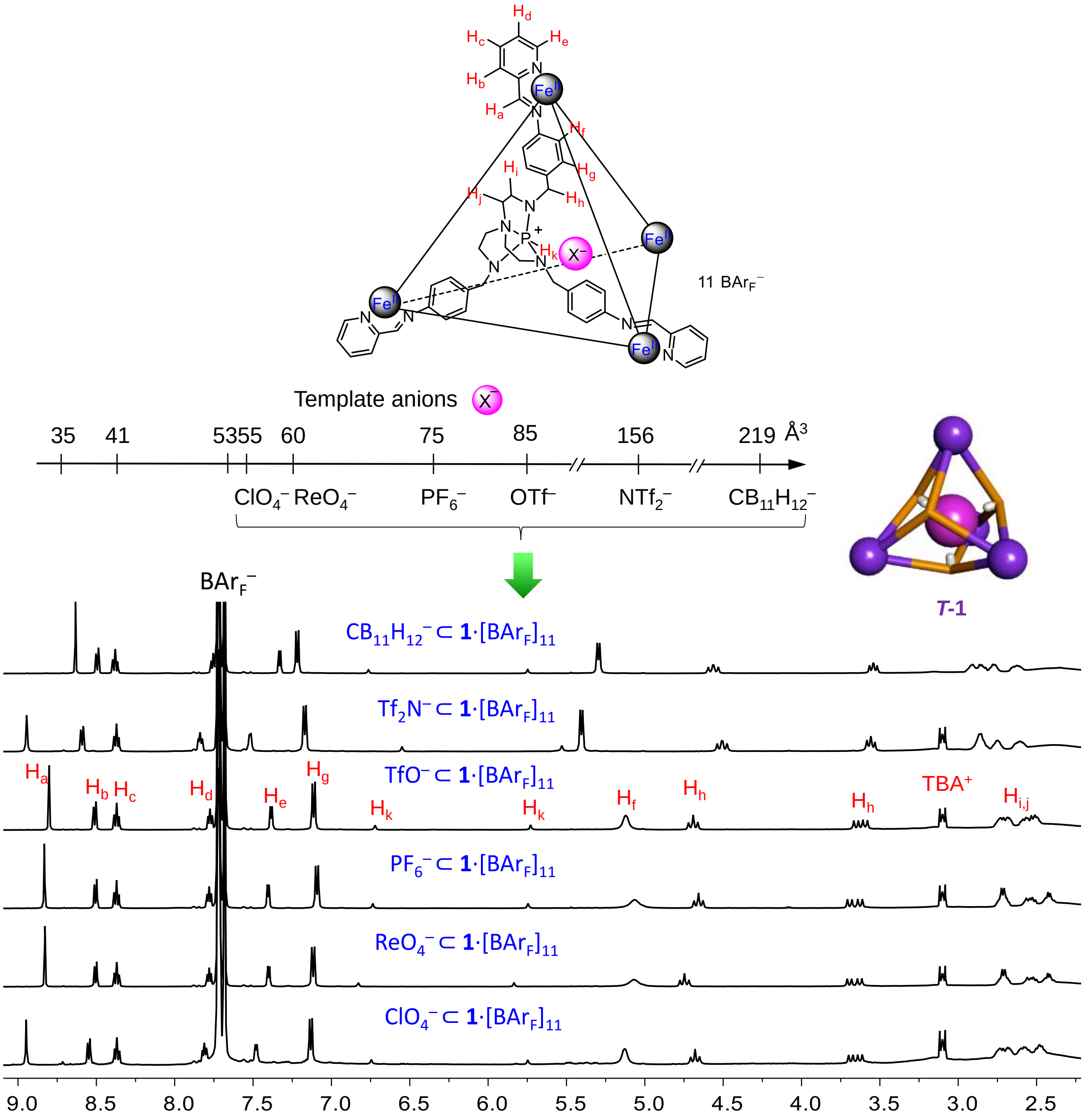

Figure S2. ^{1}H NMR (CD_3CN, 500 MHz, 298 K) spectra of the assembly of $X^-{\subset}\mathbf{1}{\cdot}[BAr_F]_{11}$ at 323 K after 16 h (X^- = ClO_4^-, ReO_4^-, PF_6^-, TfO^-, Tf_2N^- or $CB_{11}H_{12}^-$). Protons of $TfO^-{\subset}T\text{-}\mathbf{1}{\cdot}[BAr_F]_{11}$ have been assigned in the spectrum; protons assignments for cages driven by other template anions are similar and have been omitted.

2.3 Kinetic study on cage isomeric interconversion

The equation for the free energy of activation $\Delta G^{\ddagger}$ is derived in Section 2.3.1. The cage isomer interconversion was first investigated in CD_3CN at room temperature (298 K), however, it was found that the interconversion was too slow to allow calculation of $\Delta G^{\ddagger}$. Therefore, a higher temperature of 323 K was used in all cases for the kinetic investigations. The $\Delta G^{\ddagger}$ values in CD_3CN are thus higher than those in D_2O, in which interconversion could be observed at 298 K.[2]

In the case of $NO_3^-{\subset}\mathbf{1}{\cdot}[BAr_F]_{11}$, overnight experiments gave the $\Delta G^{\ddagger}$ in CD_3CN at 323 K (Figure S3 and S4). However, in the cases of $BF_4^-{\subset}\mathbf{1}{\cdot}[BAr_F]_{11}$ (Figure S5) and $I^-{\subset}\mathbf{1}{\cdot}[BAr_F]_{11}$ (Figure S6), during the interconversion, the concentration decrease of T-**1** was larger than the increase of C_3-**1**, indicating

decomposition of *T*-**1** over a period of several days. Due to this reason, these $\Delta G^{\ddagger}$ values could not be determined.

2.3.1 Equation derivation for free energy of activation $\Delta G^{\ddagger}$[2]

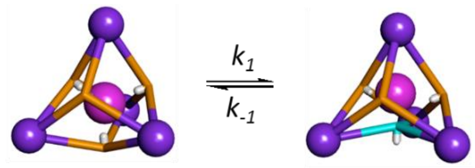

	T-1	C_3-1
t = 0	a	b
t = t	a-x	b+x

After complete formation of the cage by self-assembly (t = 0), the concentrations of *T*-**1** and C_3-**1** are "*a*" and "*b*", respectively. "k_1" and "k_{-1}" are the rate constants for the following interconversion. At any time "*t*", the concentration change, x, can be monitored by ^{1}H NMR spectroscopy. *t*-BuOH was used as the internal standard. Based on prior work,[2,3] the following equation can be obtained:

$$k_1 t = -\frac{1}{1+\frac{1}{K^0}} ln\left(1-\frac{x\left(1+\frac{1}{K^0}\right)}{a-\frac{b}{K^0}}\right)$$

Eq 1

$$K^0 = \frac{[B]^{eq}}{[A]^{eq}} = \frac{k_1}{k_{-1}}$$

Eq 2

Here "$[A]^{eq}$" and "$[B]^{eq}$" in **Eq 2** are the concentrations of two cage isomers at the equilibrium, which can be determined by ^{1}H NMR spectroscopy. Therefore, at different times "*t*", the corresponding value of "k_1t" can be calculated based on the right side of **Eq 1**. If we establish Y = $k_1 t$, Then k_1 can be determined by linear fit between Y and *t*. Based on **Eq 2**, k_{-1} could be also calculated from k_1 and K^0.

From the Eying equation, the free activation energy $\Delta G^{\ddagger}$ can be calculated as follows:

$$\Delta G^{\ddagger} = -RTln\frac{kh}{k_B T}$$

Eq 3

Here *T* is 323 K. Therefore, according to the values of k_1 and k_{-1}, the corresponding activation energies of $\Delta G_1^{\ddagger}$ and $\Delta G_{-1}^{\ddagger}$ for interconversion can be determined.

It should be noted that $\Delta G_1^{\ddagger}$ is the free energy of activation for the conversion from *T*-**1** to C_3-**1**, and k_1 is the corresponding rate constant; while $\Delta G_{-1}^{\ddagger}$ is the free energy of activation for the conversion from C_3-**1** to *T*-**1**, and k_{-1} is the corresponding rate constant.

2.3.2 Activation energy for $NO_3^- \subset$ *T*-**1** ⇌ $NO_3^- \subset$ *C*$_3$-**1**

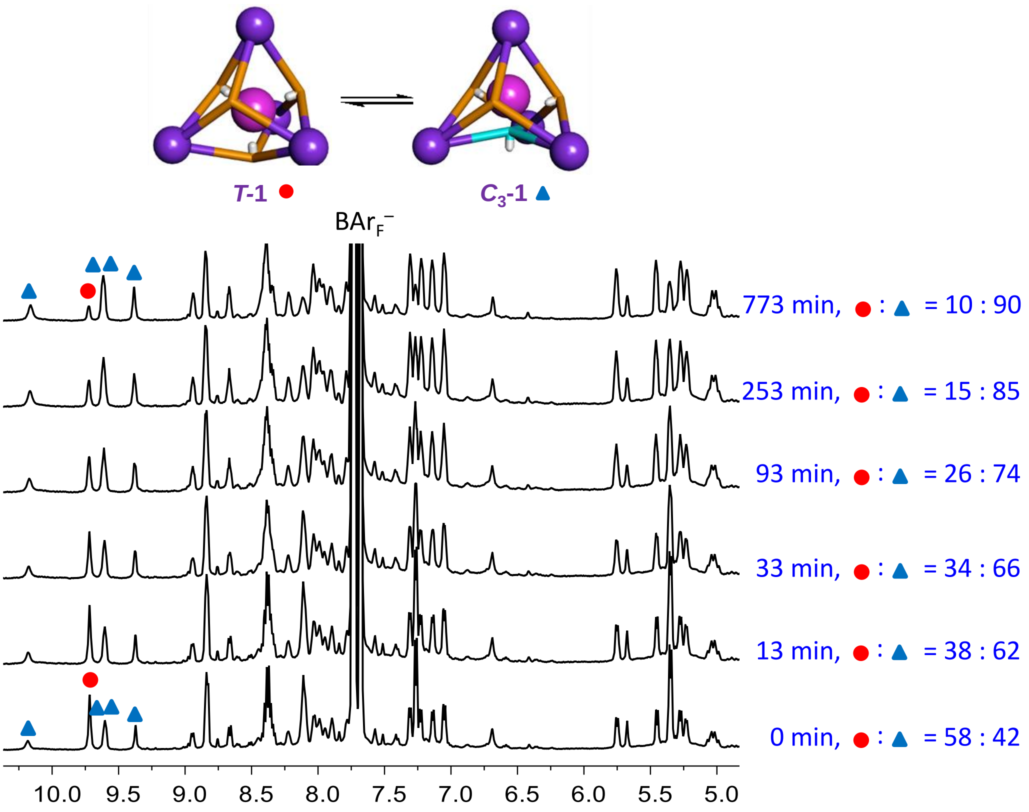

Figure S3. Time evolution of the ratio between $NO_3^- \subset$*T*-**1**·[BAr$_F$]$_{11}$ (●) and $NO_3^- \subset$*C*$_3$-**1**·[BAr$_F$]$_{11}$ (▲) at 323 K monitored by ^{1}H NMR spectroscopy (CD_3CN, 500 MHz, 323 K). Imine protons have been labelled in the spectra.

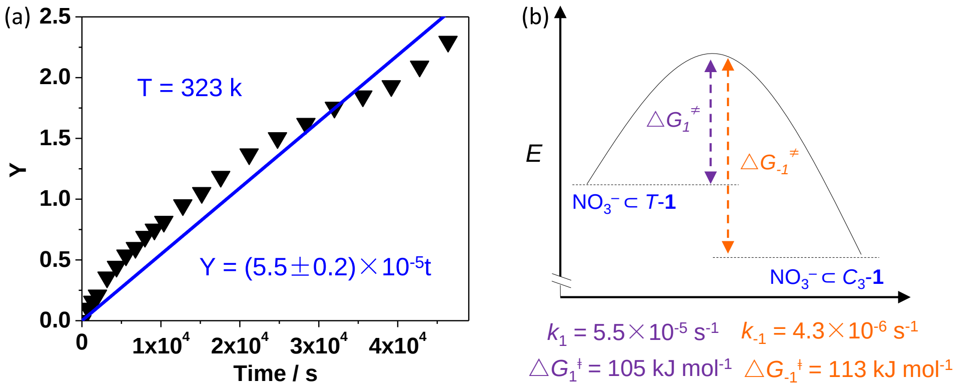

Figure S4. Kinetic study of interconversion of $NO_3^- \subset$*T*-**1**·[BAr$_F$]$_{11}$ ⇌ $NO_3^- \subset$*C*$_3$-**1**·[BAr$_F$]$_{11}$. (a) The plot between Y and the time *t*, where Y is calculated by **Eq 1** at the right side; the corresponding slope is the rate constant k_1, as indicated in Figure S4b; the time after 2 h of assembly at 50 °C (see Figure S1) was chosen as "*t* = 0"; k_{-1}, $\triangle G_1^‡$ and $\triangle G_{-1}^‡$ were determined by either **Eq 2** or **Eq 3** in Section 2.3.1. (b) Activation energy diagram of $NO_3^- \subset$*T*-**1** ⇌ $NO_3^- \subset$*C*$_3$-**1**.

2.3.3 Activation energy for $BF_4^- \subset$ ***T*-1** ⇌ $BF_4^- \subset$ ***C*₃-1**

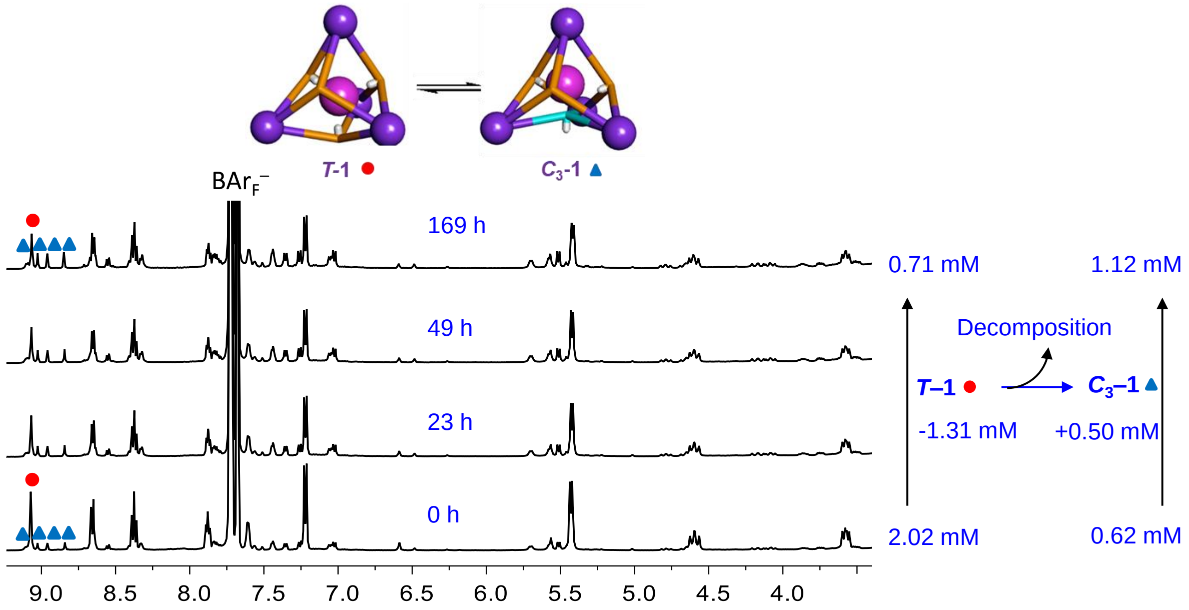

Figure S5. Time evolution of the ratio between $BF_4^-\subset T$-**1**·$[BAr_F]_{11}$ (●) and $BF_4^-\subset C_3$-**1**·$[BAr_F]_{11}$ (▲) at 323 K monitored by ^1H NMR spectroscopy (CD_3CN, 500 MHz, 298 K). Imine protons have been labelled in the spectra. During the interconversion, the concentration decrease of *T*-**1** (1.31 mM) is larger than the increase of C_3-**1** (0.50 mM), indicating the decomposition of *T*-**1** over the time period of the experiment, preventing $\triangle G^‡$ determination in this case.

2.3.4 Activation energy for $I^-\subset$ ***T*-1** ⇌ $I^-\subset$ ***C*₃-1**

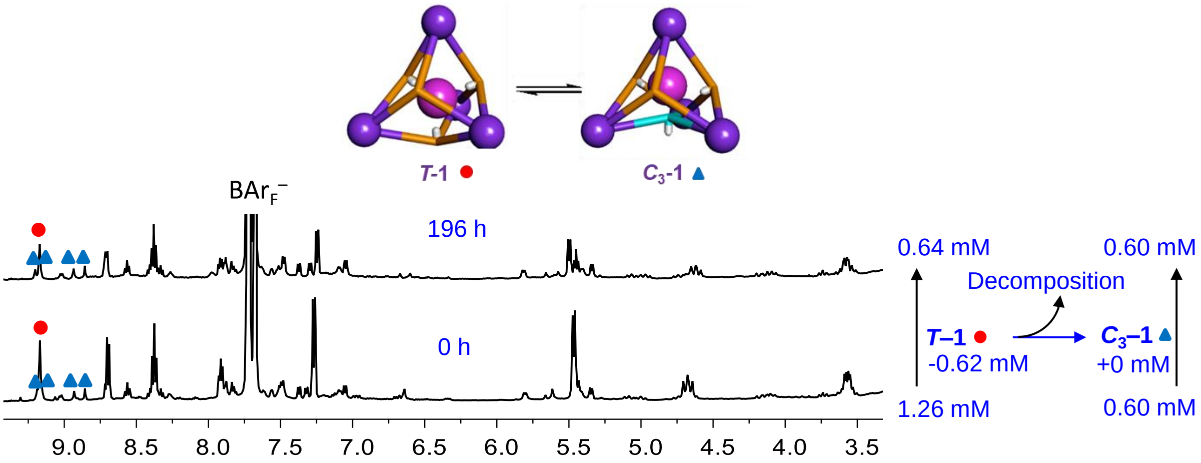

Figure S6. Time evolution of the ratio between $I^-\subset T$-**1**·$[BAr_F]_{11}$ (●) and $I^-\subset C_3$-**1**·$[BAr_F]_{11}$ (▲) at 323 K monitored by ^1H NMR spectroscopy (CD_3CN, 500 MHz, 298 K). Imine protons have been labelled in the spectra. During the interconversion, the concentration decrease of *T*-**1** (0.62 mM) is larger than the increase of C_3-**1** (0 mM), indicating the decomposition of *T*-**1** over the time period of the experiment, preventing $\triangle G^‡$ determination in this case.

2.4 Cage stability in EtOAc and CD_3NO_2

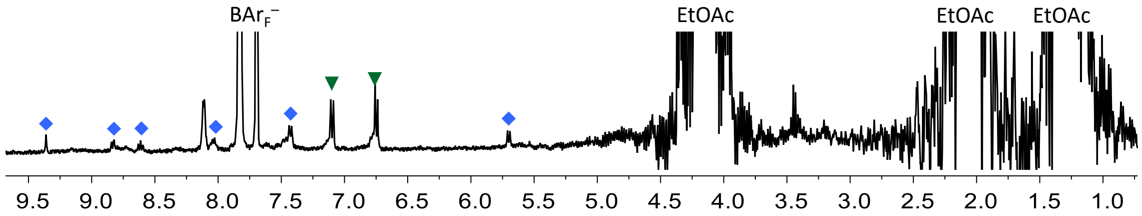

Figure S7. ^{1}H NMR spectrum (400 MHz, 298 K) of 1.5 mM Tf_2N^-⊂**1**·$[BAr_F]_{11}$ in EtOAc locked by D_2O in a capillary. Due to the unavailability of deuterated EtOAc, nondeuterated EtOAc was used, resulting in intense solvent peaks. In EtOAc, around 65% of cages disassembled to the ligands after 4 h. ◆ And ▼ represent the cage and ligand peaks, respectively.

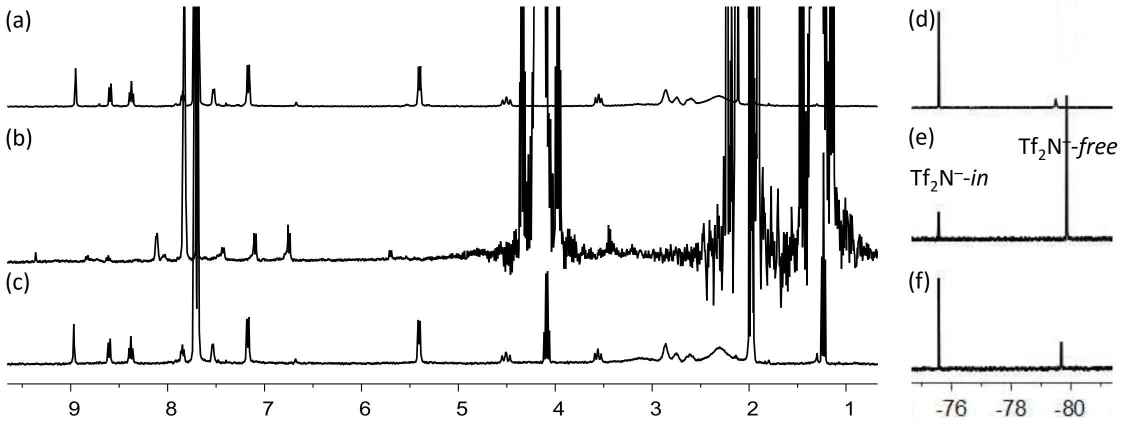

Figure S8. ^{1}H NMR spectra (400 MHz, 298 K) of 1.5 mM Tf_2N^-⊂**1**·$[BAr_F]_{11}$ (a) in CD_3CN and (b) in EtOAc. (c) ^{1}H NMR spectrum (400 MHz, 298 K) of the disassembled cage after evaporation of EtOAc and redissolving in CD_3CN at 50 °C for 16 h, which shows the recovery of the cage Tf_2N^-⊂**1**·$[BAr_F]_{11}$. ^{19}F NMR spectra (376 MHz, 298K) of 1.5 mM Tf_2N^-⊂**1**·$[BAr_F]_{11}$ (d) in CD_3CN and (e) in EtOAc. (f) ^{19}F NMR spectrum (376 MHz, 298K) of the disassembled cage after evaporation of EtOAc and redissolving in CD_3CN at 50 °C for 16 h.

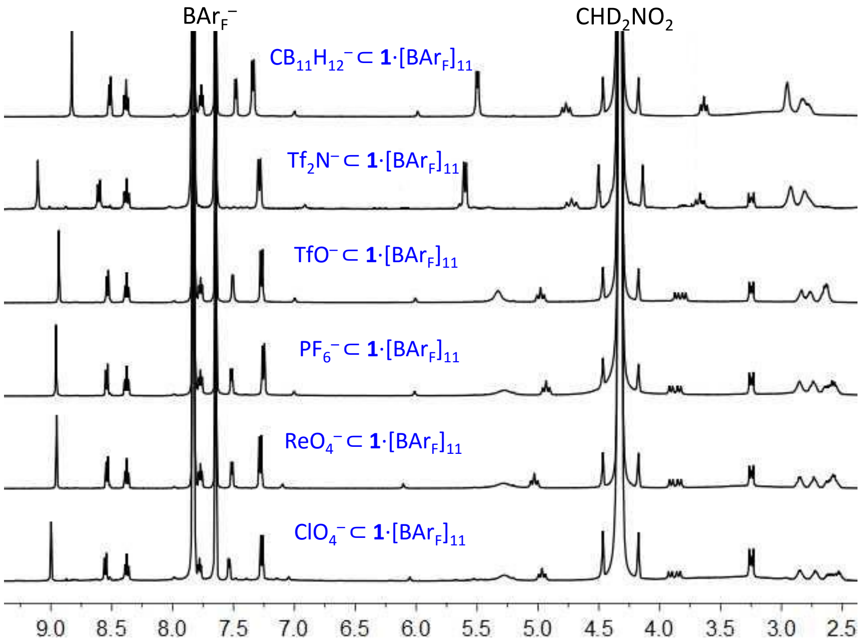

Figure S9. ^{1}H NMR (500 MHz, 298 K) spectra of 1.5 mM X^-⊂**1**·$[BAr_F]_{11}$ in CD_3NO_2 (X^- = ClO_4^-, ReO_4^-, PF_6^-, TfO^-, Tf_2N^- or $CB_{11}H_{12}^-$). No obvious changes in the spectra were observed over two weeks.

3. Competitive binding studies

A 3.0 mM stock solution of the templated cage $X^{-}\subset$**1**·$[BAr_F]_{11}$ in CD_3NO_2 was prepared through the evaporation of the initial acetonitrile solution of the cages in Section 2.2. Then its concentration was calibrated to be around 1.0 mM through dilution with CD_3NO_2, using trimethoxybenzene as an internal standard. This solution was used directly for probing the relative binding strength of anions by recording ^1H NMR spectra. In most cases, different equivalents of competitive anions were added to the reference solution in order to obtain an accurate average relative binding affinity. After each addition of the guest anion to the reference, the mixture was kept at 298 K for at least 2 days prior to recording the spectra to make sure the system had reached equilibrium.

In solution, there is the following equilibrium:

$$[HG_1] + [G_2] \rightleftarrows [HG_2] + [G_1]$$

The relative binding constant (K_{rel}) is calculated by the following equation:

$$K_{rel} = \frac{K_a^2}{K_a^1} = \frac{[HG_2]}{[HG_1]} \times \frac{[G_1]}{[G_2]}$$

Here K_{rel} is the relative binding constant; K_a^2 is the binding constant for guest **2** and K_a^1 is the binding constant for guest **1**. Prior to the addition of a competitive anion $\mathbf{G_2}$, $[G_1]_{total}$, the total concentration of $\mathbf{G_1}$, is able to be determined by integrating the peaks of TBA^+ relative to the trimethoxybenzene standard. Due to the slow exchange between the host and guest on the NMR time scale, after addition of $\mathbf{G_2}$, $[HG_1]$ and $[HG_2]$ could be determined by integration of the corresponding cage imine peak relative to the ^1H NMR standard. Then $[G_1]$, the concentration of the free $\mathbf{G_1}$, is equal to the difference between $[G_1]_{total}$ and $[HG_1]$. The concentration of the added $\mathbf{G_2}$, $[G_2]_{add}$, could be also accurately determined by the increase of the TBA^+ integrations relative to the standard, and then $[G_2]$, the concentration of free $\mathbf{G_2}$, is equal to the difference between $[G_2]_{add}$ and $[HG_2]$. Hence based on the above four concentrations, the relative binding constant K_{rel} can be determined by ^1H NMR spectroscopy.

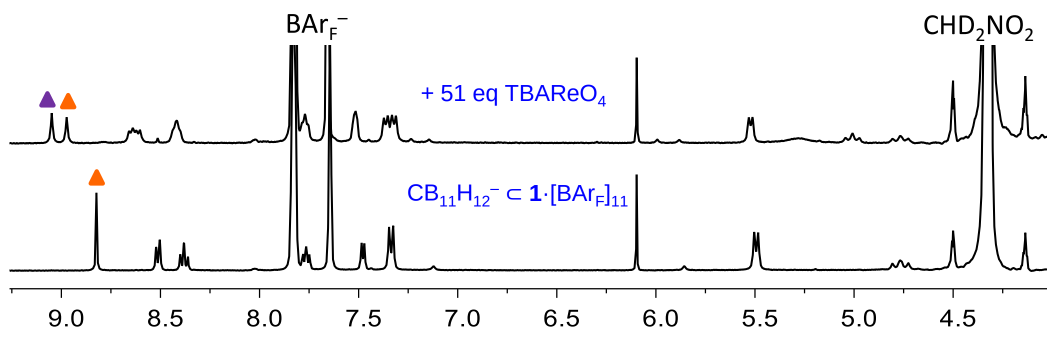

Figure S10. Addition of ReO_4^- to a solution of $CB_{11}H_{12}^{-}\subset$**1**·$[BAr_F]_{11}$ in CD_3NO_2 monitored by ^1H NMR spectroscopy (400 MHz, 298K). The solution was equilibrated for 2 days following addition of ReO_4^- prior to acquisition of the new spectrum. The results show $K_{CB11H12-} = 52K_{ReO4-}$. The downfield shift of the initial imine peak of $CB_{11}H_{12}^{-}\subset$**1**·$[BAr_F]_{11}$ after addition of ReO_4^- indicates exterior interactions with the cage.[4]

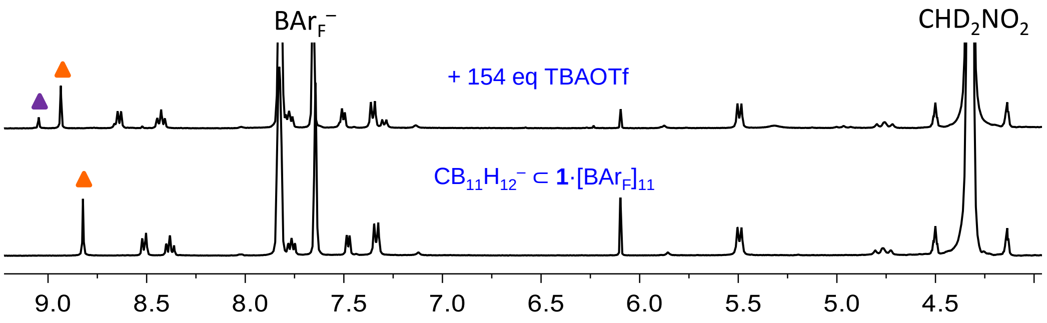

Figure S11. Addition of TfO^- to a solution of $CB_{11}H_{12}^- \subset$**1**·$[BAr_F]_{11}$ in CD_3NO_2 monitored by ^1H NMR spectroscopy (400 MHz, 298K). The solution was equilibrated for 2 days following addition of TfO^- prior to acquisition of the new spectrum. The results show $K_{CB11H12-} = 1240K_{TfO-}$. The downfield shift of the initial imine peak of $CB_{11}H_{12}^- \subset$**1**·$[BAr_F]_{11}$ after addition of TfO^- indicates exterior interactions with the cage.[4]

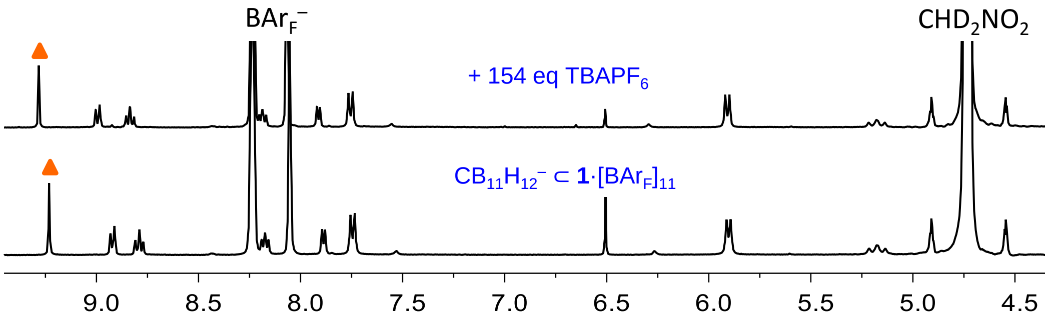

Figure S12. Addition of PF_6^- to a solution of $CB_{11}H_{12}^- \subset$**1**·$[BAr_F]_{11}$ in CD_3NO_2 monitored by ^1H NMR spectroscopy (400 MHz, 298K). The solution was equilibrated for 2 days following addition of PF_6^- prior to acquisition of the new spectrum. The results show that PF_6^- was unable to displace the encapsulated $CB_{11}H_{12}^-$ due to its too weaker binding affinity. The downfield shift of the imine peak of $CB_{11}H_{12}^- \subset$**1**·$[BAr_F]_{11}$ after adding PF_6^- indicates exterior interactions with the cage.[4]

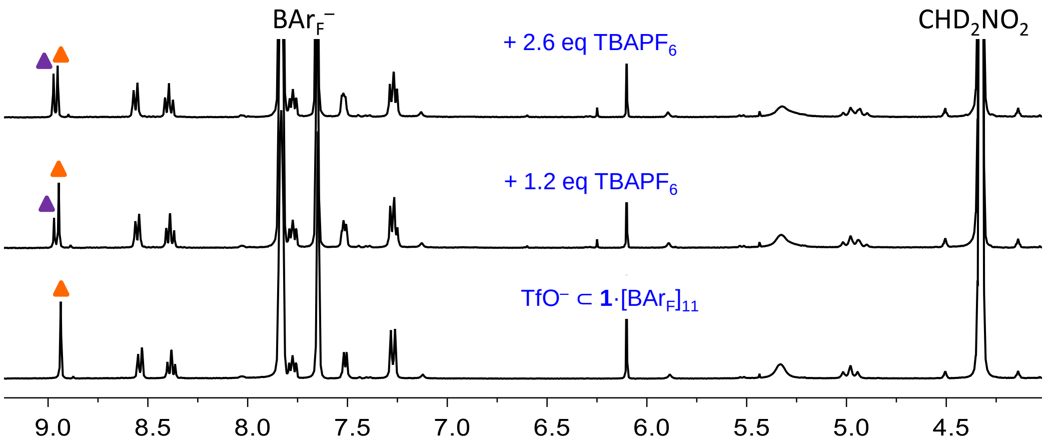

Figure S13. Addition of PF_6^- to a solution of $TfO^- \subset$**1**·$[BAr_F]_{11}$ in CD_3NO_2 monitored by ^1H NMR spectroscopy (400 MHz, 298K). The solution was equilibrated for 2 days following addition of PF_6^- prior to acquisition of the new spectrum. The results show $K_{TfO-} = 2.8K_{PF6-}$.

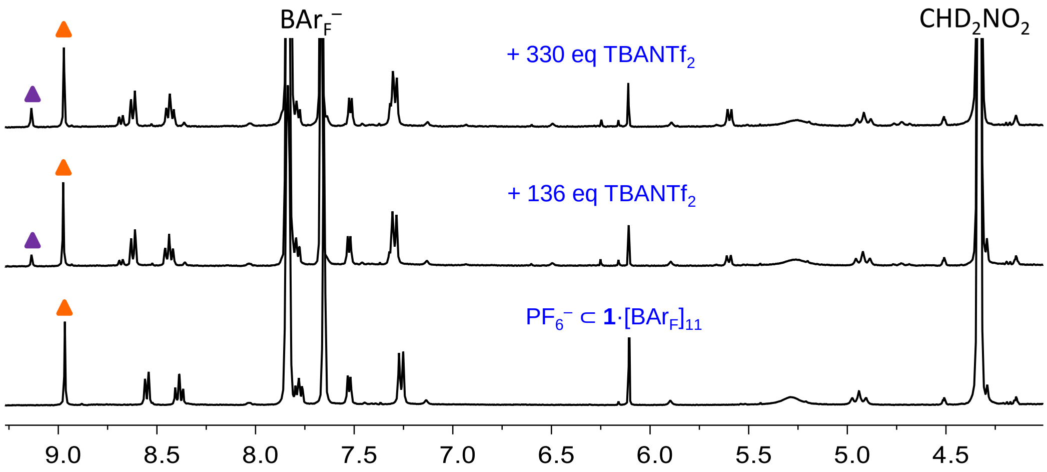

Figure S14. Addition of Tf_2N^- to a solution of $PF_6^- \subset \mathbf{1} \cdot [BAr_F]_{11}$ in CD_3NO_2 monitored by ^1H NMR spectroscopy (400 MHz, 298K). The solution was equilibrated for 2 days following addition of Tf_2N^- prior to acquisition of the new spectrum. The results show $K_{PF6-} = 872K_{Tf2N-}$.

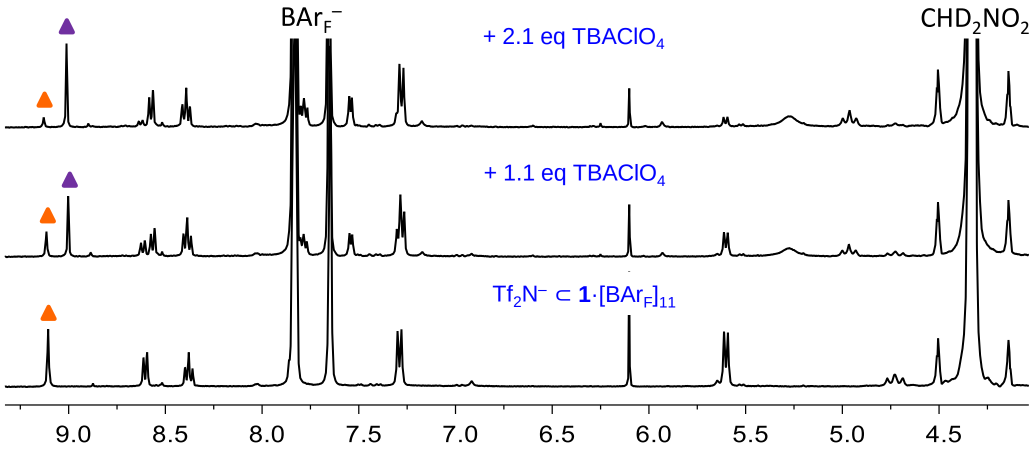

Figure S15. Addition of ClO_4^- to a solution of $Tf_2N^- \subset \mathbf{1} \cdot [BAr_F]_{11}$ in CD_3NO_2 monitored by ^1H NMR spectroscopy (400 MHz, 298K). The solution was equilibrated for 2 days following addition of ClO_4^- prior to acquisition of the new spectrum. The results show $K_{ClO4-} = 6.8K_{Tf2N-}$.

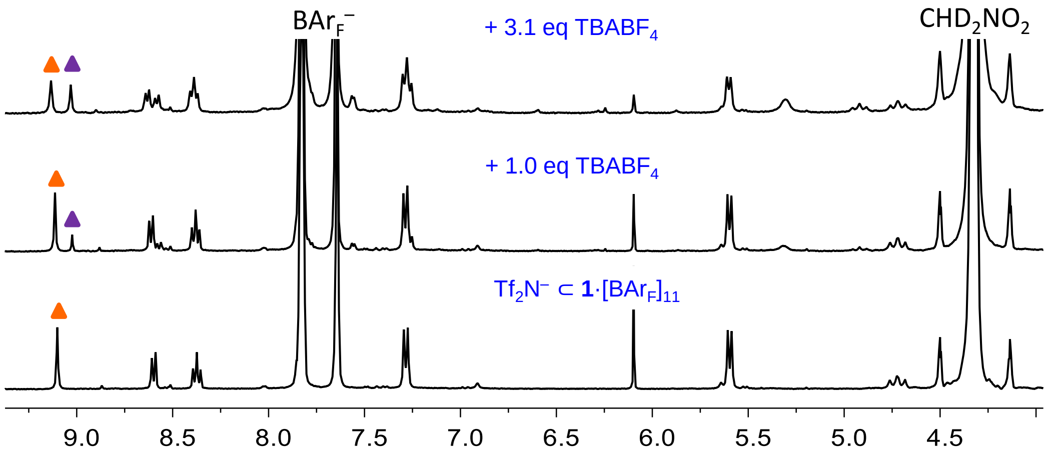

Figure S16. Addition of BF_4^- to a solution of $Tf_2N^- \subset \mathbf{1} \cdot [BAr_F]_{11}$ in CD_3NO_2 monitored by ^1H NMR spectroscopy (400 MHz, 298K). The solution was equilibrated for 2 days following addition of BF_4^- prior to acquisition of the new spectrum. The results show $K_{Tf2N-} = 4.7K_{BF4-}$.

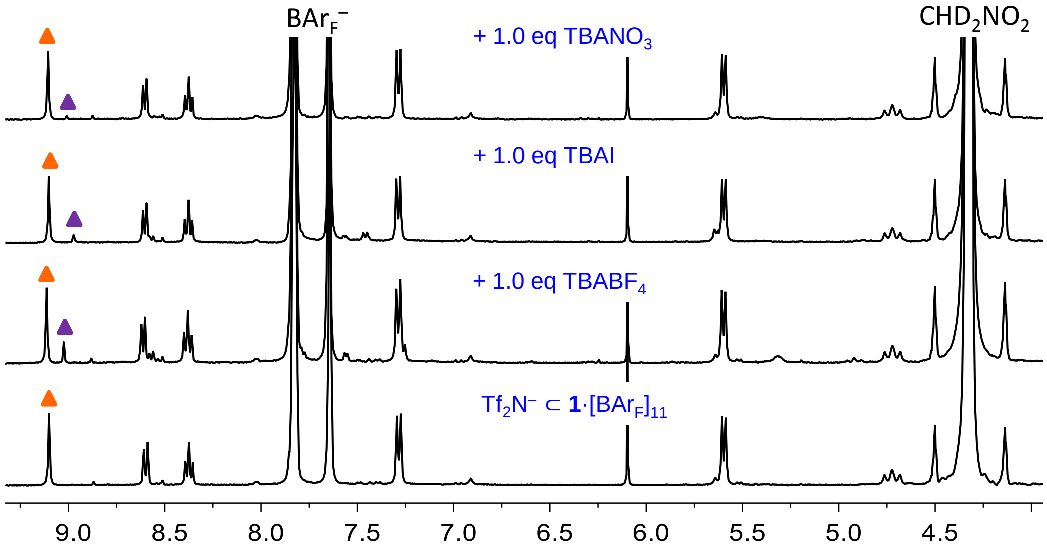

Figure S17. Addition of 1 equiv of BF_4^-, I^- or NO_3^- to a solution of $Tf_2N^- \subset$**1**·[BAr$_F$]$_{11}$ in CD_3NO_2 monitored by ^{1}H NMR spectroscopy (400 MHz, 298K). The solution was equilibrated for 2 days following addition of the anionic guest prior to acquisition of the new spectrum. The results show that 1 equiv of NO_3^- or I^- gave rise to a new set of peaks with too low intensity to allow calculation of the relative binding constants; while larger amounts caused the decomposition of the cage. Nevertheless, from the comparison of the intensities of the new peaks, a binding order of $BF_4^- > I^- > NO_3^-$ could be elucidated.

From Figures S10-S13, an order of binding affinities, $CB_{11}H_{12}^- > ReO_4^- > TfO^- > PF_6^-$, could be derived. From Figures S15-S17, an order of binding affinities, $ClO_4^- > Tf_2N^- > BF_4^- > I^- > NO_3^-$, could be determined. Combined with the result of Figure S14, the relative binding affinities of all the different anions in CD_3NO_2 could be established: $CB_{11}H_{12}^- > ReO_4^- > TfO^- > PF_6^- > ClO_4^- > Tf_2N^- > BF_4^- > I^- > NO_3^-$. Table S1 shows relative binding constants of the different anions compared to Tf_2N^- in CD_3NO_2.

Table S1. Relative binding constants of the different anions compared to Tf_2N^- in CD_3NO_2.

Anion	Tf_2N^-	$CB_{11}H_{12}^-$	ReO_4^-	TfO^-	PF_6^-	ClO_4^-	BF_4^-	I^-	NO_3^-
Relative binding affinity	1	3.0×10^6	5.8×10^4	2.4×10^3	8.7×10^2	6.8	2.1×10^{-1}	—[a]	—[a]

[a] *Relative binding constant could not be calculated due to the new peaks having insufficient intensity during ^{1}H NMR titrations.*

4. X-ray crystallography

Data were collected either using a Bruker D8 VENTURE equipped with high-brilliance IμS Cu-Kα radiation (1.54178 Å), with ω and ψ scans at 180(2) K or at Beamline I19 of Diamond Light Source employing silicon double crystal monochromated synchrotron radiation (0.6889 Å) with ω and ψ scans at 100(2) K. Data integration and reduction were undertaken with SAINT and XPREP[5] or with Xia2.[6] Subsequent computations were carried out using the WinGX-32 graphical user interface.[7] Multi-scan empirical absorption corrections were applied to the data using SADABS[5] or the AIMLESS[8] tool in the CCP4 suite.[9] Structures were solved by direct methods using SHELXT[10] then refined and extended with SHELXL.[11] In general, non-hydrogen atoms with occupancies greater than 0.5 were refined anisotropically. Carbon-bound hydrogen atoms were included in idealised positions and refined using a riding model. Disorder was modelled using standard crystallographic methods including constraints, restraints and rigid bodies where necessary. Crystallographic data along with specific details pertaining to the refinement follow. Crystallographic data have been deposited with the CCDC (1812469-1812470).

[$CB_{11}H_{12}$⊂1]·9.15$CB_{11}H_{12}$·1.85NTf_2·8.25MeCN·Et_2O·0.5H_2O

Formula $C_{214.35}H_{341.55}B_{111.65}F_{11.10}Fe_4N_{50.10}O_{8.90}P_4S_{3.70}$, *M* 5646.64, Monoclinic, space group P 21/n (#14), *a* 23.9461(2), *b* 43.9741(2), *c* 28.49200(10) Å, *β* 91.4190(10), *V* 29993.1(3) Å^3, D_c 1.250 g cm^{-3}, *Z* 4, crystal size 0.005 by 0.004 by 0.004 mm, color purple, habit block, temperature 100(2) Kelvin, *λ*(Synchrotron) 0.6889 Å, *μ*(Synchrotron) 0.278 mm^{-1}, T(Analytical)$_{min,max}$ 0.947445441606, 1.0, $2\theta_{max}$ 48.42, *hkl* range -27 27, -52 52, -33 33, *N* 205354, N_{ind} 51744(R_{merge} 0.0356), N_{obs} 36099(I > 2 σ(I)), N_{var} 4130, residuals* *R*1(*F*) 0.0893, *wR*2(F^2) 0.2920, GoF(all) 1.082, $\Delta\rho_{min,max}$ -1.283, 1.530 e$^-$ Å^{-3}.

*$R1 = \Sigma||F_o| - |F_c||/\Sigma|F_o|$ for $F_o > 2\sigma(F_o)$; $wR2 = (\Sigma w(F_o^2 - F_c^2)^2/ \Sigma(wF_c^2)^2)^{1/2}$ all reflections, $w=1/[\sigma^2(F_o^2)+(0.1850P)^2+20.3170P]$ where $P=(F_o^2+2F_c^2)/3$

Specific refinement details:

Crystals of [$CB_{11}H_{12}$⊂**1**]·9.15$CB_{11}H_{12}$·1.85NTf_2·8.25MeCN·Et_2O·0.5H_2O were grown by diffusion of diethyl ether into an acetonitrile solution of a mixture of **1**·12NTf_2 and $CsCB_{11}H_{12}$. The crystals employed immediately lost solvent after removal from the mother liquor and rapid handling prior to flash cooling in the cryostream was required to collect data. The asymmetric unit was found to contain one complete Fe_4L_4 assembly and associated counterions and solvent molecules. Phosphorus-bound hydrogen atoms were first located in the difference Fourier map before refinement.

Due to the presence of thermal motion within the structure, bond lengths and angles within pairs of organic ligands were restrained to be similar to each other (SAME) and thermal parameter restraints (SIMU, RIGU) were applied to all atoms except for iron. Two anion lattice sites within the structure show disorder between Tf_2N^- and $CB_{11}H_{12}^-$ anions. All Tf_2N^- anions within the structure show evidence of substantial disorder and were modelled as disordered over two or three locations. Substantial bond length and thermal parameter restraints were applied to facilitate a reasonable refinement of the disordered Tf_2N^- anions. Where anion disorder was observed the occupancies of the disordered parts were refined to sum to one and later fixed at the obtained values. The $CB_{11}H_{12}^-$ anions also show evidence of disorder with one $CB_{11}H_{12}^-$ anion modelled as disordered over two discrete locations and several others showing evidence of rotational disorder and/or thermal motion. For these anions where the carbon site could not be clearly discerned from the electron density map all atoms of the disordered $CB_{11}H_{12}^-$ were modelled as boron. Even in cases where the carbon site could be identified, some additional rotational disorder cannot be ruled out. All $CB_{11}H_{12}^-$ anions were restrained to have a similar icosahedral geometry (SAME). Several acetonitrile solvent molecules were also modelled as disordered over multiple locations with bond length restraints.

[ReO_4⊂1]·6ReO_4·5NTf_2·4.83MeCN·0.5Et_2O·0.33H_2O [+ solvent]

Formula $C_{201.67}H_{204.17}F_{30}Fe_4N_{49.83}O_{48.83}P_4Re_7S_{10}$, *M* 6648.58, orthorhombic, space group P 2_1 2_1 2_1 (#19), *a* 41.014(2), *b* 41.490(2), *c* 48.117(3) Å, *V* 81880(7) Å^3, D_c 1.618 g cm^{-3}, *Z* 12, crystal size 0.190 by 0.150 by 0.120 mm, colour purple, habit prism, temperature 180(2) Kelvin, λ(CuKα) 1.54178 Å, μ(CuKα) 9.296 mm^{-1}, T(SADABS)$_{min,max}$ 0.5940, 0.7500, $2\theta_{max}$ 101.37, *hkl* range -33 41, -41 40, -46 48, *N* 326796, N_{ind} 85243(R_{merge} 0.0607), N_{obs} 63563(I > 2σ(I)), N_{var} 9956, residuals* $R1(F)$ 0.0854, $wR2(F^2)$ 0.2519, GoF(all) 1.023, $\Delta\rho_{min,max}$ -1.301, 3.158 e$^-$ Å^{-3}.

*$R1 = \Sigma||F_o| - |F_c||/\Sigma|F_o|$ for $F_o > 2\sigma(F_o)$; $wR2 = (\Sigma w(F_o^2 - F_c^2)^2/ \Sigma(wF_c^2)^2)^{1/2}$ all reflections, $w=1/[\sigma^2(F_o^2)+(0.1521P)^2+806.9008P]$ where $P=(F_o^2+2F_c^2)/3$

Specific refinement details:

Crystals of [ReO_4⊂**1**]·6ReO_4·5NTf_2·4.83MeCN·0.5Et_2O·0.33H_2O were grown by diffusion of diethyl ether into an acetonitrile solution of a mixture of **1**·12NTf_2 and TBAReO_4. The crystals employed immediately lost solvent after removal from the mother liquor and rapid handling prior to flash cooling in the cryostream was required to collect data. Despite these measures and the use of a high intensity laboratory source few reflections at greater than 1.0 Å resolution were observed. Nevertheless, the quality of the data is more than sufficient to establish the connectivity of the structure. Due to the limited resolution of the data the phosphorus-bound hydrogen atoms could not be located in the Fourier map and were therefore included in idealised positions and refined using a riding model. The asymmetric unit was found to contain three complete Fe_4L_4 assemblies and associated counterions and solvent molecules.

Due to the presence of thermal motion within the structure, bond lengths and angles within pairs of organic ligands were restrained to be similar to each other (SAME) and thermal parameter restraints (SIMU, RIGU) were applied to all atoms except for iron and rhenium. The anions within the structure show evidence of substantial disorder. The oxygen atoms of the three encapsulated ReO_4^- anions were modelled as disordered over two or three locations with overall occupancies of 100% each. Many of the other ReO_4^- anions and Tf_2N^- anions were also modelled as disordered over multiple locations and/or with partial occupancy. Some additional minor occupancy positions of these anions could not be located in the electron density map due to the limited resolution of the data. One anion lattice site was modelled as a disordered mixture of both ReO_4^- and Tf_2N^-. Where anion disorder was observed the occupancies of the disordered parts were refined and later fixed at the obtained values. Substantial bond length and thermal parameter restraints were applied to facilitate a reasonable refinement of the disordered anions. Several solvent molecules were also modelled as disordered over multiple locations with bond length restraints. Hydrogen atoms were not modelled for some of these disordered solvents.

Further reflecting the solvent loss and poor diffraction properties there is a significant amount of void volume in the lattice containing smeared electron density from further disordered solvent and up to 1.5 unresolved anions per Fe_4L_4 complex. Consequently the SQUEEZE[12] function of PLATON[13] was employed to remove the contribution of the electron density associated with this highly disordered solvent.

5. Volume calculations

In order to determine the available void space within **1** in the complexes ReO_4^-⊂**1** and $CB_{11}H_{12}^-$⊂**1**, VOIDOO calculations[14] based on the crystal structures were performed. For ReO_4^-⊂**1** a virtual probe with a radius of 1.4 Å (set by default, water-sized) was employed while the more open structure of $CB_{11}H_{12}^-$⊂**1** required a probe with a radius of 1.8 Å as smaller probes were observed to exit the cavity during the calculation. The standard parameters tabulated below were used in both cases, following the previously published procedure.[15]

Maximum number of volume-refinement cycles:	30
Minimum size of secondary grid:	3
Grid for plot files:	0.1
Primary grid spacing:	0.1

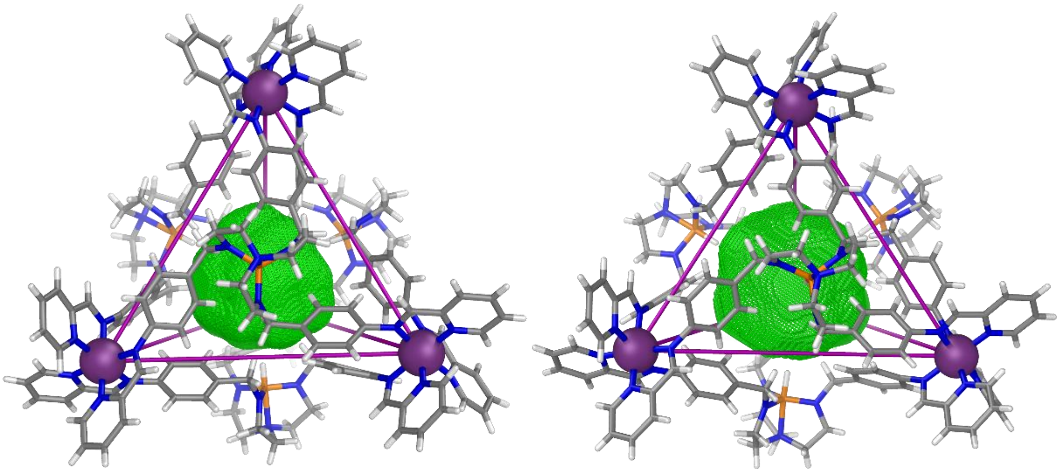

Figure S18. VOIDOO-calculated void space as shown (green mesh) within the crystal structures of ReO_4^-⊂**1** (left, volume: 157 Å^3) and $CB_{11}H_{12}^-$⊂**1** (right, volume: 253 Å^3) in the absence of the encapsulated anions. For ReO_4^-⊂**1**, only one of the three crystallographically unique cages is shown and the calculated volume is given as the average value for all three cages.

6. Anion extraction experiments

6.1 Anion extraction by $Tf_2N^-\subset$1·[BArF]11

Liquid-liquid extractions using $Tf_2N^- \subset$ **1**·$[BAr_F]_{11}$ as the extractant were undertaken as follows: 0.8 mM $Tf_2N^- \subset$ **1**·$[BAr_F]_{11}$ in 600 μl CD_3NO_2 and 0.8 mM $NaReO_4$/NaOTf in 600 μl D_2O were placed in a vial. The two phases were mixed throughly by stirring at rt. After 7 h, the stirring was stopped and the vial was allowed to stand for 30 min to fully separate the two phases. The ^1H NMR and ^{19}F NMR spectra of each phase were then recorded. Trimethoxybenzene was used as an internal standard in CD_3NO_2. To investigate the effect of concentration, extraction experiments were carried out at other concentrations of $Tf_2N^- \subset$ **1**·$[BAr_F]_{11}$, such as 0.6 mM and 1.3 mM .

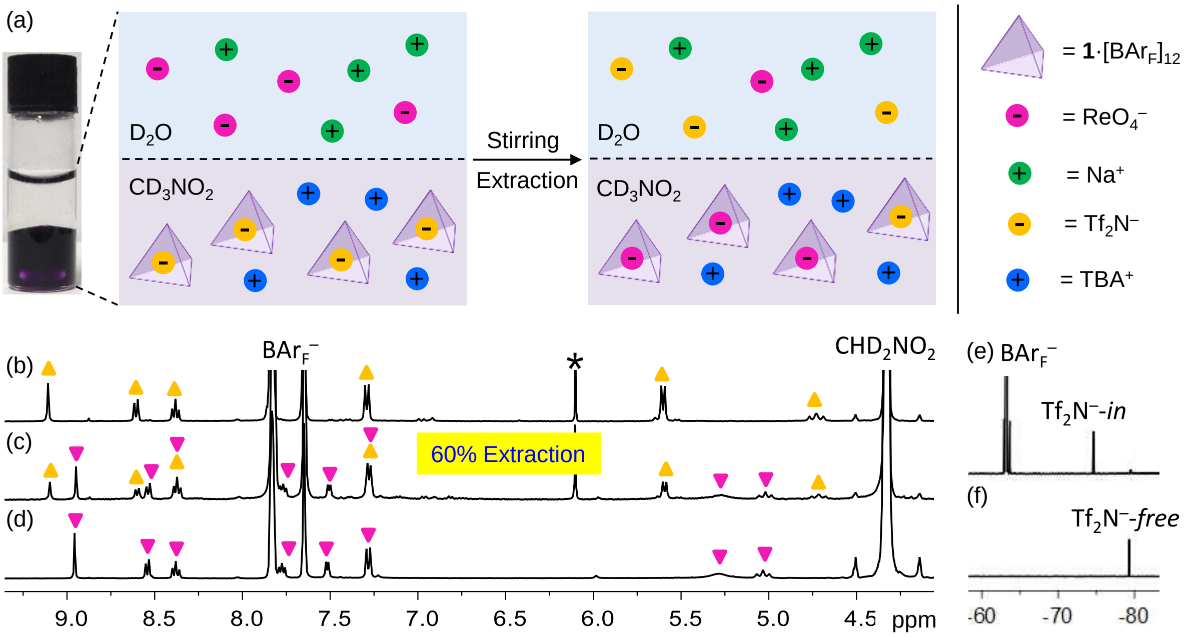

Figure S19. (a) Illustration of the liquid-liquid extraction of ReO_4^- from water to an organic phase (conditions: 0.8 mM $Tf_2N^- \subset$ **1**·$[BAr_F]_{11}$ in 600 μl CD_3NO_2; 0.8 mM $NaReO_4$ in 600 μl D_2O; 7 hours of stirring at rt). Partial ^1H NMR spectra (400 MHz, 298K) of (b) the CD_3NO_2 phase before extraction, showing only the presence of $Tf_2N^- \subset$ **1**·$[BAr_F]_{11}$ (▲), (c) the CD_3NO_2 phase after extraction, showing the presence of 40% $Tf_2N^- \subset$ **1**·$[BAr_F]_{11}$ (▲) and 60% $ReO_4^- \subset$ **1**·$[BAr_F]_{11}$ (▼), and (d) $ReO_4^- \subset$ **1**·$[BAr_F]_{11}$ in CD_3NO_2 (▼). ^{19}F NMR spectra (376 MHz, 298K) of (e) the CD_3NO_2 phase after extraction, showing the presence of BAr_F^-, encapsulated Tf_2N^- and a trace of free Tf_2N^-, and (f) the D_2O phase after extraction, showing the presence of free Tf_2N^-. The trimethoxybenzene standard is marked with an asterisk.

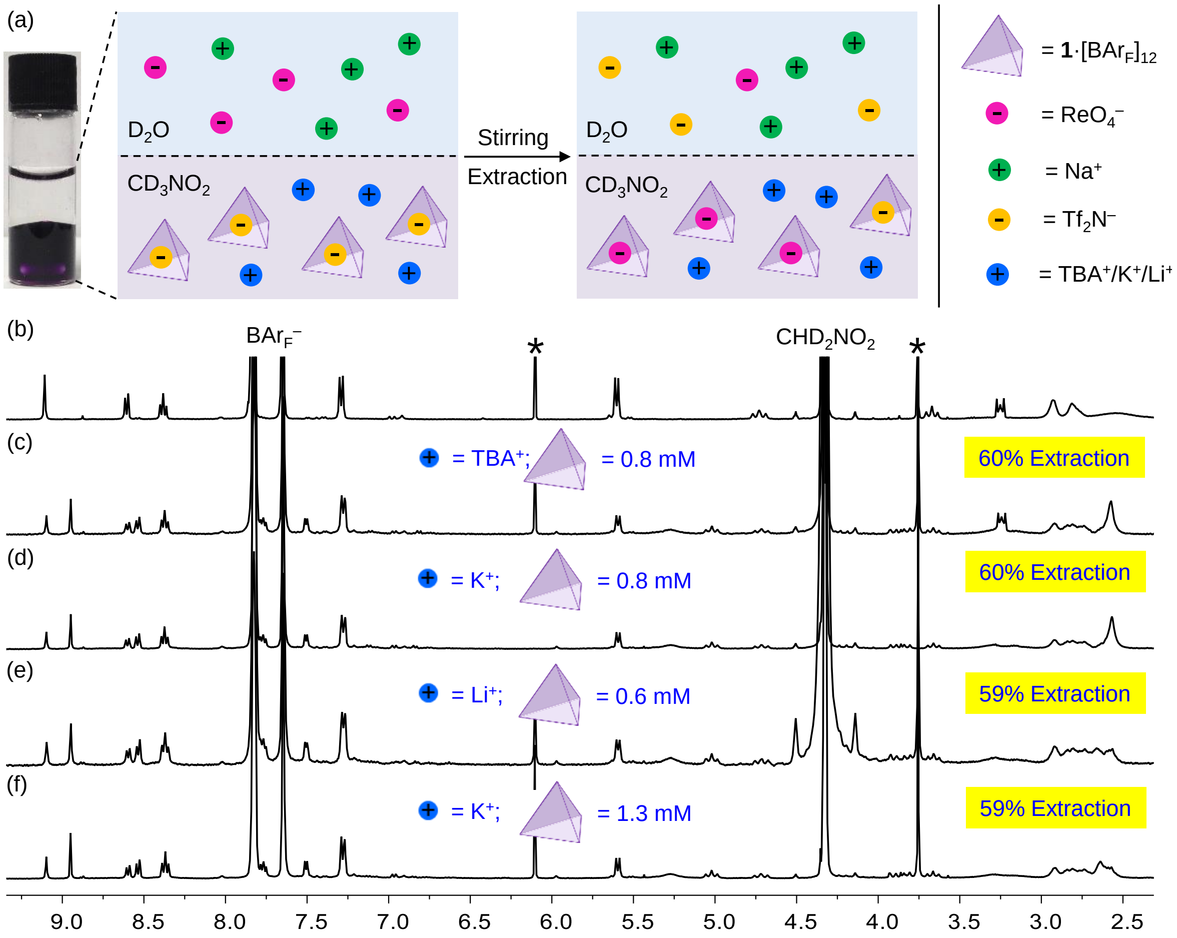

Figure S20. (a) Illustration of the liquid-liquid extraction of ReO_4^- from water to an organic phase. Partial ^1H NMR spectra (400 MHz, 298K) of (b) the CD_3NO_2 phase before extraction, showing only the presence of $Tf_2N^- \subset$ **1**·$[BAr_F]_{11}$, (c) the CD_3NO_2 phase after extraction when using $TBANTf_2$ as the source of the template (conditions: 0.8 mM $Tf_2N^- \subset$ **1**·$[BAr_F]_{11}$ in 600 µl CD_3NO_2; 0.8 mM $NaReO_4$ in 600 µl D_2O; 7 hours of stirring at rt), (d) the CD_3NO_2 phase after extraction when using $KNTf_2$ as the source of the template (conditions: 0.8 mM $Tf_2N^- \subset$ **1**·$[BAr_F]_{11}$ in 600 µl CD_3NO_2; 0.8 mM $NaReO_4$ in 600 µl D_2O; 7 hours of stirring at rt), (e) the CD_3NO_2 phase after extraction when using $LiNTf_2$ as the source of the template (conditions: 0.6 mM $Tf_2N^- \subset$ **1**·$[BAr_F]_{11}$ in 600 µl CD_3NO_2; 0.6 mM $NaReO_4$ in 600 µl D_2O; 7 hours of stirring at rt), and (f) the CD_3NO_2 phase after extraction when using $KNTf_2$ as the source of the template (conditions: 1.3 mM $Tf_2N^- \subset$ **1**·$[BAr_F]_{11}$ in 600 µl CD_3NO_2; 1.3 mM $NaReO_4$ in 600 µl D_2O; 7 hours of stirring at rt). The trimethoxybenzene standard is marked with an asterisk.

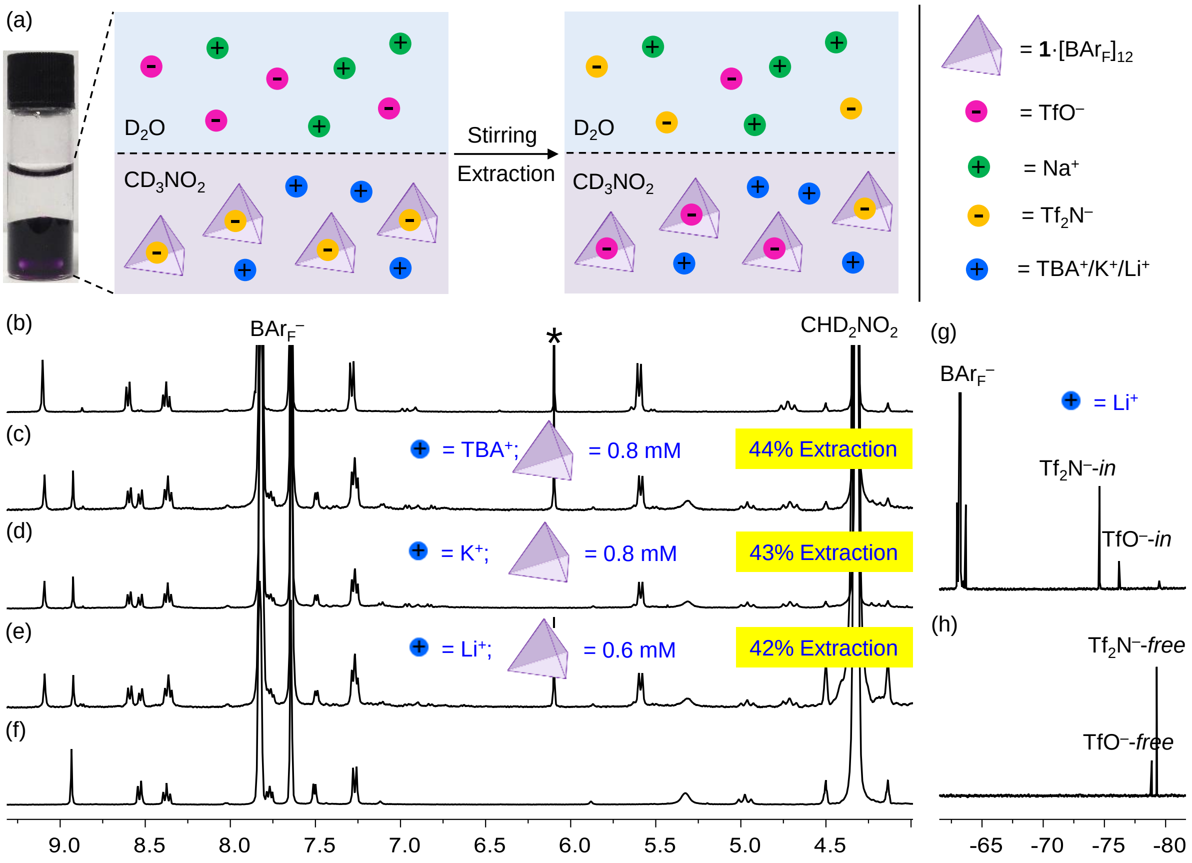

Figure S21. (a) Illustration of the liquid-liquid extraction of TfO⁻ from water to an organic phase. Partial ^{1}H NMR spectra (400 MHz, 298K) of (b) the CD_3NO_2 phase before extraction, showing only the presence of $Tf_2N^- \subset$ **1**·$[BAr_F]_{11}$, (c) the CD_3NO_2 phase after extraction when using $TBANTf_2$ as the source of the template (conditions: 0.8 mM $Tf_2N^- \subset$ **1**·$[BAr_F]_{11}$ in 600 µl CD_3NO_2; 0.8 mM NaOTf in 600 µl D_2O; 7 hours of stirring at rt), (d) the CD_3NO_2 phase after extraction when using $KNTf_2$ as the source of the template (conditions: 0.8 mM $Tf_2N^- \subset$ **1**·$[BAr_F]_{11}$ in 600 µl CD_3NO_2; 0.8 mM NaOTf in 600 µl D_2O; 7 hours of stirring at rt), (e) the CD_3NO_2 phase after extraction when using $LiNTf_2$ as the source of the template (conditions: 0.6 mM $Tf_2N^- \subset$ **1**·$[BAr_F]_{11}$ in 600 µl CD_3NO_2; 0.6 mM NaOTf in 600 µl D_2O; 7 hours of stirring at rt), and (f) $TfO^- \subset$ **1**·$[BAr_F]_{11}$ in CD_3NO_2. ^{19}F NMR spectra (376 MHz, 298K) of (e) the CD_3NO_2 phase after extraction, showing the presence of BAr_F^-, encapsulated Tf_2N^-, encapsulated TfO^- and a trace of free Tf_2N^-, and (f) the D_2O phase after extraction, showing the presence of free Tf_2N^- and TfO^-. The trimethoxybenzene standard is marked with an asterisk.

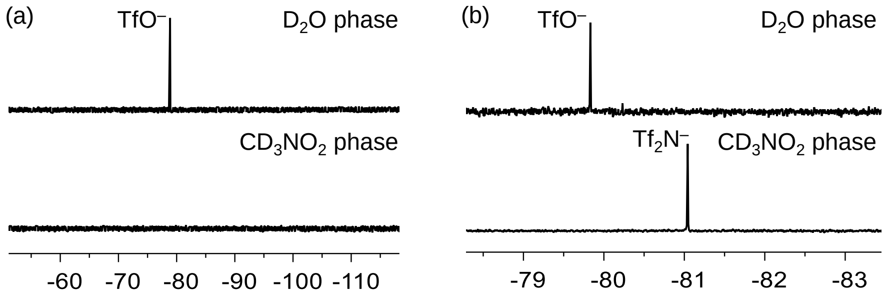

Figure S22. (a) Distribution of NaOTf between two phases (conditions: 600 µl pure CD_3NO_2; 0.8 mM NaOTf in 600 µl D_2O). ^{19}F NMR spectra (376 MHz, 298K) of the D_2O phase (upper) and CD_3NO_2 phase (bottom) after 7 hours of stirring at rt. (b) Distribution of NaOTf between two phases in the presence of $TBANTf_2$ (conditions: 0.8 mM $TBANTf_2$ in 600 µl CD_3NO_2; 0.8 mM NaOTf in 600 µl D_2O). ^{19}F NMR spectra (376 MHz, 298K) of the D_2O phase (upper) and CD_3NO_2 phase (bottom) after 7 hours of stirring at rt.

6.2 Anion extraction by $^{n}BuBF_3^{-}\subset$**1**·$[BAr_F]_{11}$

6.2.1 Cage formation driven by the $^{n}BuBF_3^{-}$ template

The preparation of $^{n}BuBF_3^{-}\subset$**1**·$[BAr_F]_{11}$ was carried out following the procedures described in Section 2.2. The salt $^{n}BuBF_3K$ was used as the template.

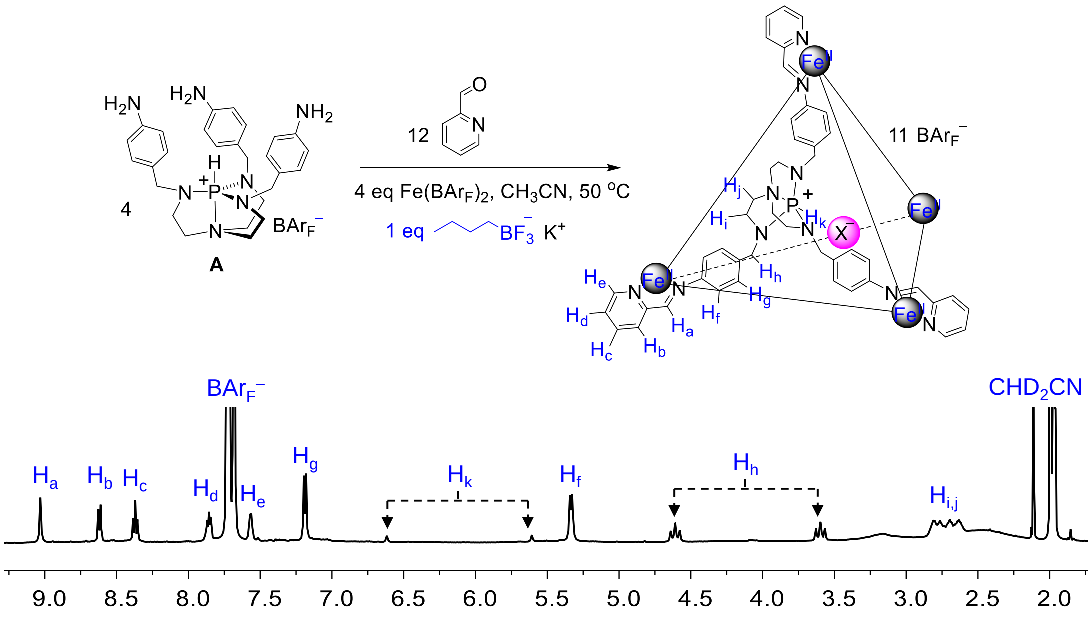

Figure S23. ^{1}H NMR spectrum (CD_3CN, 500 MHz, 298 K) of the assembly of $^{n}BuBF_3^{-}\subset$**1**·$[BAr_F]_{11}$ at 323 K for 16 h.

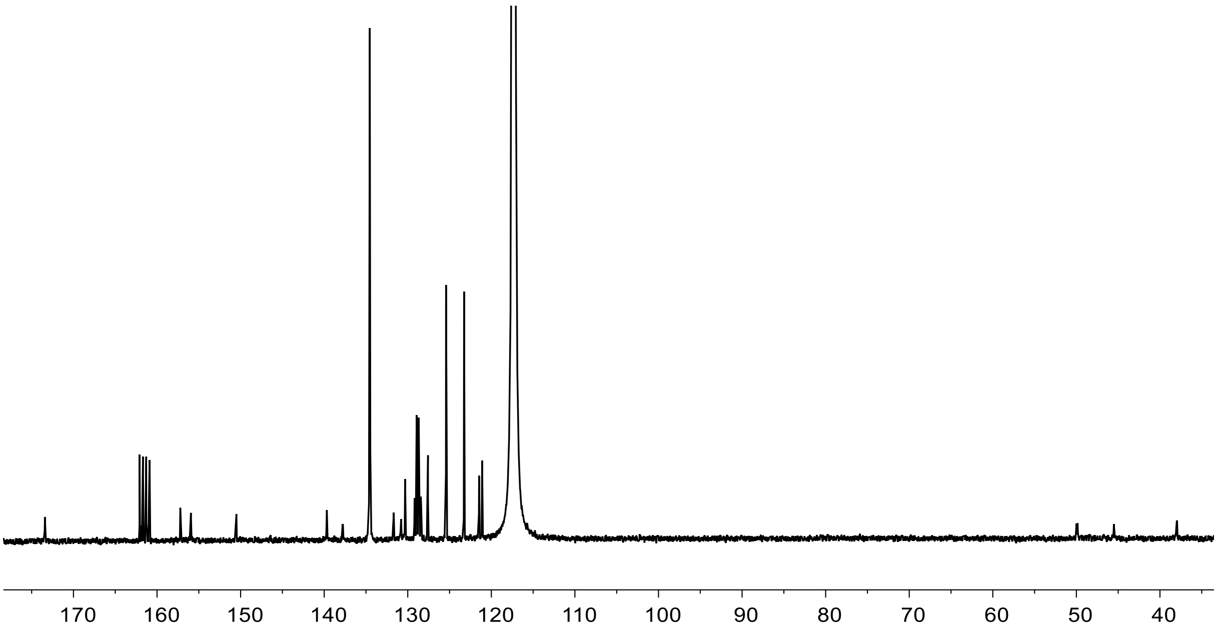

Figure S24. ^{13}C NMR spectrum (CD_3CN, 125.8 MHz, 298K) of $^{n}BuBF_3^{-}\subset$**1**·$[BAr_F]_{11}$.

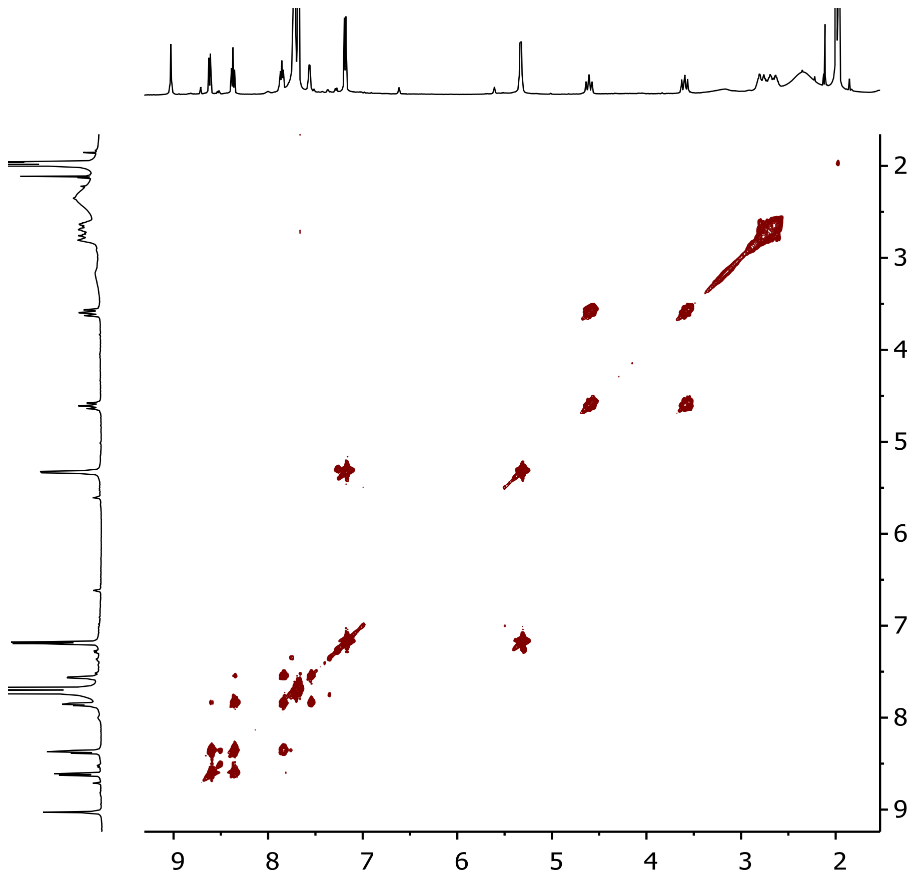

Figure S25. ^1H-^1H COSY spectrum (CD_3CN, 500 MHz, 298K) of $^nBuBF_3^- \subset \mathbf{1}\cdot[BAr_F]_{11}$.

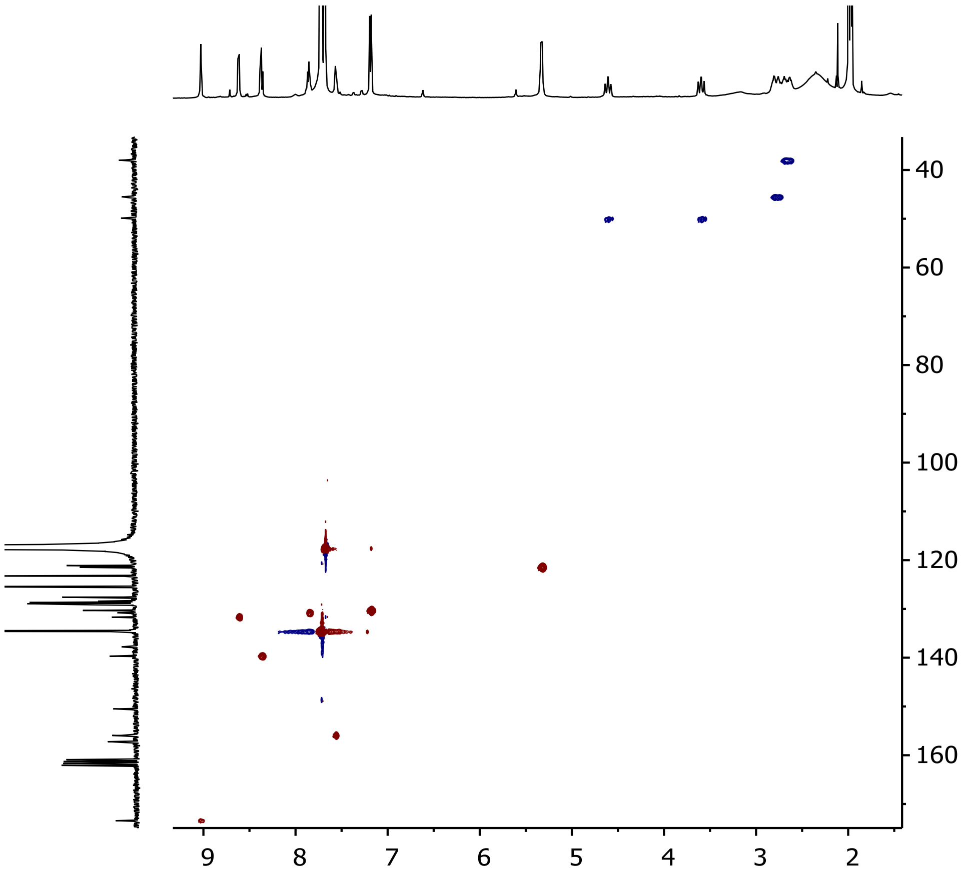

Figure S26. ^1H-^{13}C HSQC spectrum (CD_3CN, 500 MHz, 298K) of $^nBuBF_3^- \subset \mathbf{1}\cdot[BAr_F]_{11}$.

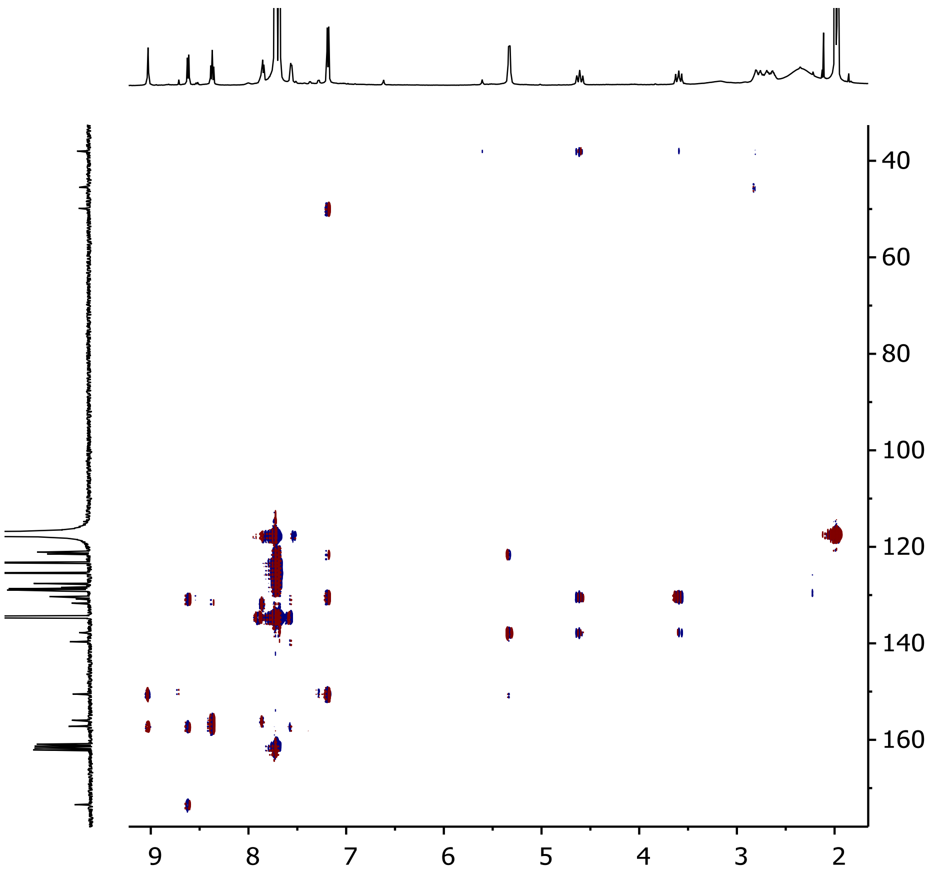

Figure S27. ^{1}H-^{13}C HMBC spectrum (CD_3CN, 500 MHz, 298K) of $^{n}BuBF_3^{-}\subset\mathbf{1}\cdot[BAr_F]_{11}$.

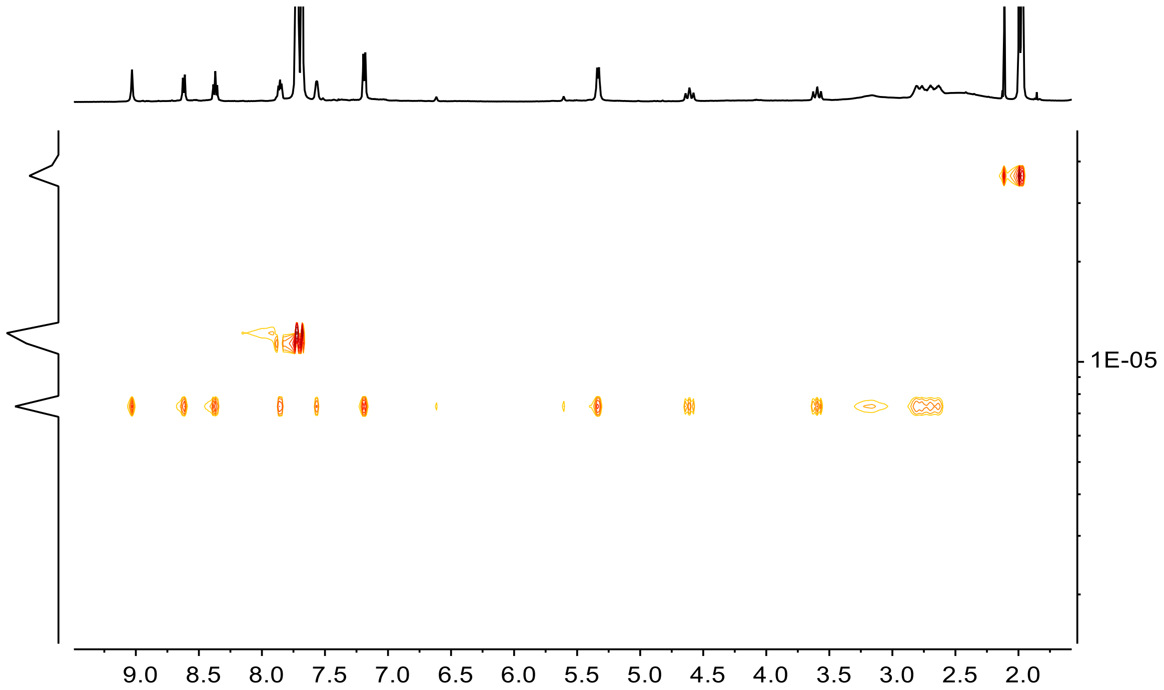

Figure S28. ^{1}H DOSY spectrum (CD_3CN, 500 MHz, 298K) of $^{n}BuBF_3^{-}\subset\mathbf{1}\cdot[BAr_F]_{11}$. The diffusion coefficient of $^{n}BuBF_3^{-}\subset\mathbf{1}\cdot[BAr_F]_{11}$ in CD_3CN was measured to be 7.34×10^{-6} cm^2 s^{-1}.

6.2.2 Binding affinity of $^{n}BuBF_3^-$ relative to Tf_2N^-

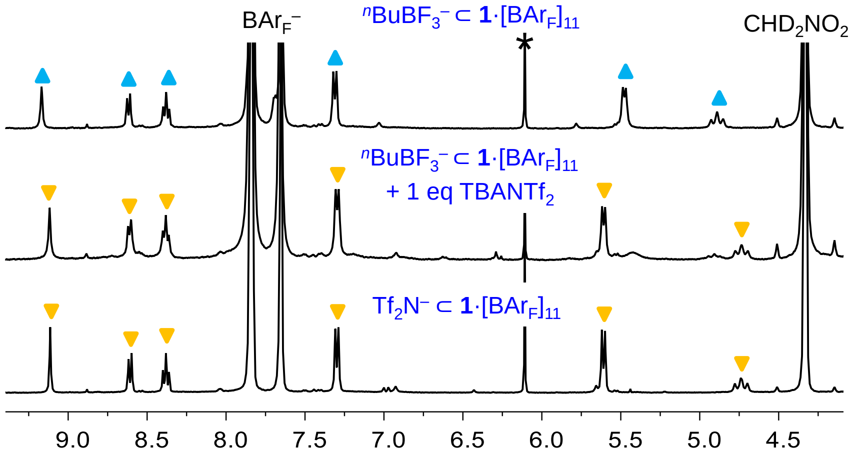

Figure S29. Addition of Tf_2N^- to a solution of $^{n}BuBF_3^- \subset$ **1**·$[BAr_F]_{11}$ in CD_3NO_2 monitored by ^1H NMR (400 MHz, 298K) spectroscopy. The solution was equilibrated for 2 days following addition of $^{n}BuBF_3^-$ prior to acquisition of the new spectrum. The results show that 1 equiv of Tf_2N^- almost completely displaced the encapsulated $^{n}BuBF_3^-$. The trimethoxybenzene standard is marked with an asterisk.

6.2.3 Anion extraction by $^{n}BuBF_3^- \subset$1·$[BAr_F]_{11}$

Liquid-liquid extractions using $^{n}BuBF_3^- \subset$ **1**·$[BAr_F]_{11}$ as the extractant were undertaken as follows: 0.8 mM $^{n}BuBF_3^- \subset$ **1**·$[BAr_F]_{11}$ in 600 µl CD_3NO_2 and 0.8 mM $NaReO_4$/NaOTf in 600 µl D_2O were placed in a vial. The two phases were mixed throughly by stirring at rt. After 7 h, the stirring was stopped and the vial was allowed to stand for 30 min to fully separate the two phases. The ^1H NMR and/or ^{19}F NMR spectra of each phase were then recorded. Trimethoxybenzene was used as an internal standard in CD_3NO_2.

ReO_4^- extraction selectivity was tested as follows: 0.8 mM $^{n}BuBF_3^- \subset$ **1**·$[BAr_F]_{11}$ in 600 µl CD_3NO_2, 0.8 mM $NaReO_4$ and 10 competing anions, comprising 0.8 mM in each of NaF, NaCl, NaBr, NaI, Na_2SO_4, $KClO_4$, KNO_3, $NaBF_4$, NaH_2PO_4, and NaOAc in 600 µl D_2O were placed in a vial. The two phases were mixed throughly by stirring at rt. After 7 h, the stirring was stopped and the vial was allowed to stand for 30 min to fully separate the two phases. The ^1H NMR spectra of each phase were then recorded. Trimethoxybenzene was used as an internal standard in CD_3NO_2.

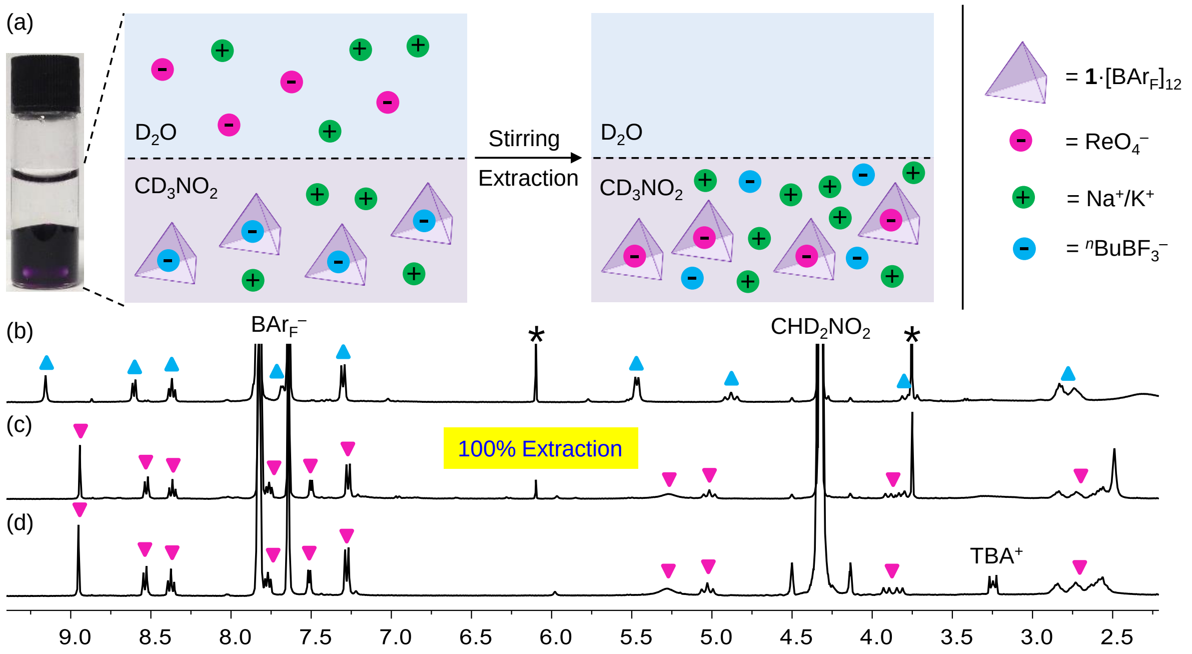

Figure S30. (a) Illustration of the liquid-liquid extraction of ReO_4^- from water to an organic phase (conditions: 0.8 mM ^{n}BuBF$_3^-$ ⊂ **1**·[BAr$_F$]$_{11}$ in 600 µl CD_3NO_2; 0.8 mM $NaReO_4$ in 600 µl D_2O; 7 hours of stirring at rt). Partial ^{1}H NMR spectra (400 MHz, 298K) of (b) the CD_3NO_2 phase before extraction, showing only the presence of ^{n}BuBF$_3^-$ ⊂ **1**·[BAr$_F$]$_{11}$ (▲), (c) the CD_3NO_2 phase after extraction, showing only the presence of ReO_4^- ⊂ **1**·[BAr$_F$]$_{11}$ (▼), and (d) ReO_4^- ⊂ **1**·[BAr$_F$]$_{11}$ in CD_3NO_2 (▼). The trimethoxybenzene standard is marked with an asterisk.

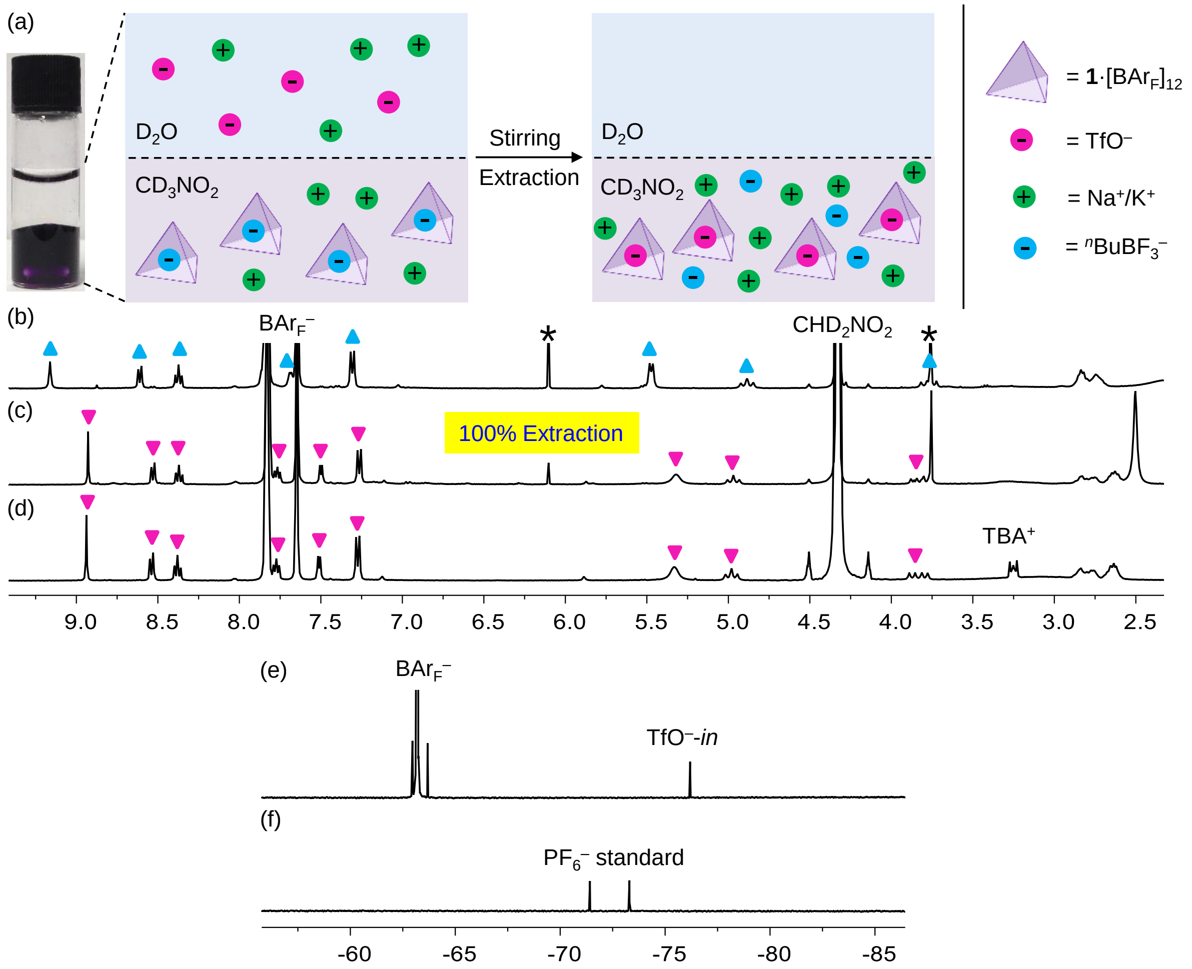

Figure S31. (a) Illustration of the liquid-liquid extraction of TfO$^-$ from water to an organic phase (conditions: 0.8 mM ^{n}BuBF$_3^-$ ⊂ **1**·[BAr$_F$]$_{11}$ in 600 μl CD_3NO_2; 0.8 mM NaOTf in 600 μl D_2O; 7 hours of stirring at rt). Partial ^{1}H NMR spectra (400 MHz, 298K) of (b) the CD_3NO_2 phase before extraction, showing only the presence of ^{n}BuBF$_3^-$ ⊂ **1**·[BAr$_F$]$_{11}$ (▲), (c) the CD_3NO_2 phase after extraction, showing only the presence of TfO$^-$ ⊂ **1**·[BAr$_F$]$_{11}$ (▼), and (d) TfO$^-$ ⊂ **1**·[BAr$_F$]$_{11}$ in CD_3NO_2 (▼). Partial ^{19}F NMR spectra (376 MHz, 298K) of (e) the CD_3NO_2 phase after extraction, showing the presence of BAr$_F^-$ and the encapsulated TfO$^-$, and (f) the D_2O phase after extraction, showing that except for the post-added PF_6^- standard, there is no TfO$^-$. The trimethoxybenzene standard is marked with an asterisk.

6.3 Cage extractant recycle

As shown in Figure S32a, initially around 1 mL, 0.8 mM ReO_4^-/TfO⁻ ⊂ **1**·[BAr_F]$_{11}$ in CD_3NO_2 was evaporated. The resulting purple solid was redissolved in 4 mL EtOAc, and 1 mL H_2O was also added to the same vial. After throuhly mixing the two phases for 3 h by stirring at rt under an inert atmosphere, the cage disassembled in EtOAc, giving rise to a light purple solution. The guest was released from the cage and transferred to the H_2O phase. This was demonstrated by ^{19}F NMR when starting from TfO⁻ ⊂ **1**·[BAr_F]$_{11}$, with the ^{19}F NMR signal from free TfO⁻ observed in the spectra of the H_2O layer (Figure 32e). Replacing the aqueous phase with 1 mL fresh water and stirring for another 1 h rendered complete separation of the guest from the disassembled cage in EtOAc. It should be noted that all the procedures above were carried out in a glovebox to provide an inert atmosphere and all the solvents used were degassed. The EtOAc layer was then separated and the solvent was evaporated. The obtained purple solid was redissolved in 500 µl CD_3CN together with 1 equiv of potassium *n*-butyltrifluoroborate with regard to the initial cage concentration. After heating at 50 °C for 16 h, the cage extractant $^nBuBF_3^-$ ⊂ **1**·[BAr_F]$_{11}$ was recovered (Figures S32b and S32c).

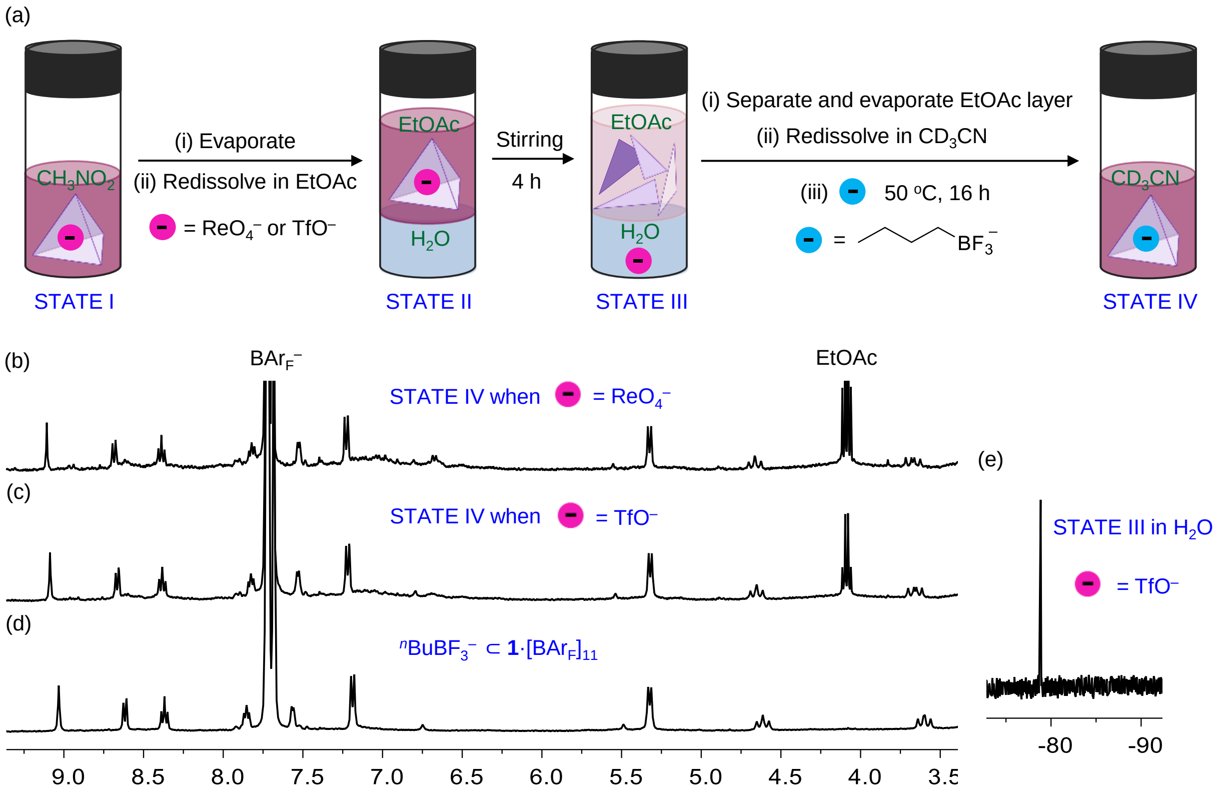

Figure S32. (a) Illustration of the strategy for cage extractant recycle. Partial ^1H NMR spectra (400 MHz, 298K) of (b) the CD_3CN solution in STATE IV, which shows the spectrum of the recycled $^nBuBF_3^-$ ⊂ **1**·[BAr_F]$_{11}$ when the initial encapsulated anion in STATE I is ReO_4^-, (c) the CD_3CN solution in STATE IV , which shows the spectrum of the recycled $^nBuBF_3^-$ ⊂ **1**·[BAr_F]$_{11}$ when the initial encapsulated anion in STATE I is TfO⁻, and (d) $^nBuBF_3^-$ ⊂ **1**·[BAr_F]$_{11}$ in CD_3CN. (e) ^{19}F NMR spectrum (376 MHz, 298K) of the H_2O phase in STATE III when the initial encapsulated anion in STATE I is TfO⁻, showing the presence of TfO⁻ species.

6.4 Anion extraction from an organic phase to water using $Tf_2N^- \subset$ 1·$[SO_4]_{5.5}$

Liquid-liquid extractions using $Tf_2N^- \subset$ **1**·$[SO_4]_{5.5}$ as the extractant were undertaken as follows: 0.8 mM $Tf_2N^- \subset$ **1**·$[SO_4]_{5.5}$ in 600 µl D_2O and 0.8 mM $TBAReO_4$/TBAOTf in 600 µl CD_3NO_2 were placed in a vial. The two phases were mixed throughly by stirring at rt. After 3 h, the stirring was stopped and the vial was allowed to stand for 30 min to fully separate the two phases. The ^1H NMR and ^{19}F NMR spectra of each phase were then recorded.

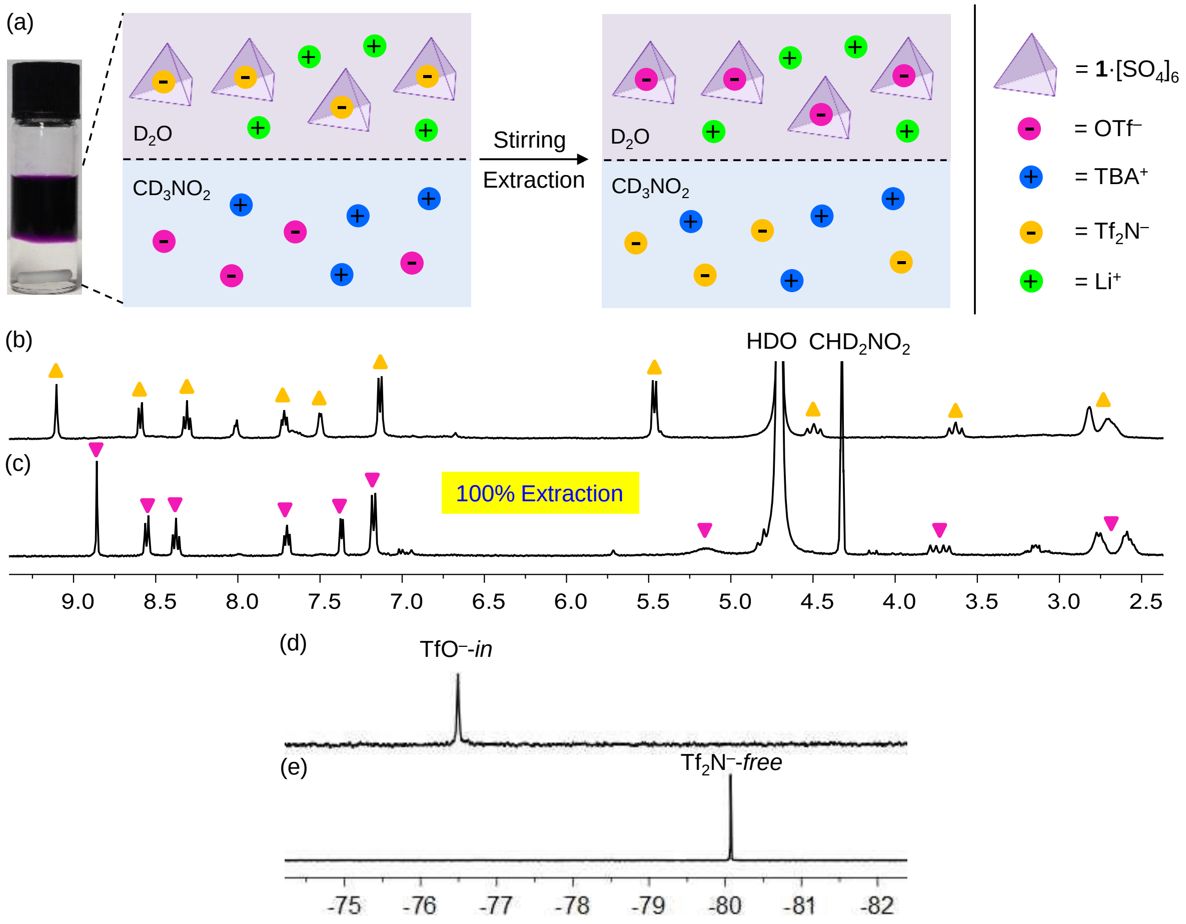

Figure S33. (a) Illustration of the liquid-liquid extraction of TfO^- from an organic phase to water (conditions: 0.8 mM $Tf_2N^- \subset$ **1**·$[SO_4]_{5.5}$ in 600 µl D_2O; 0.8 mM TBAOTf in 600 µl CD_3NO_2; 3 hours of stirring at rt). Partial ^1H NMR spectra (400 MHz, 298K) of (b) the D_2O phase before extraction, showing only the presence of $Tf_2N^- \subset$ **1**·$[SO_4]_{5.5}$ (▲), and (c) the D_2O phase after extraction, showing only the presence of $TfO^- \subset$ **1**·$[BAr_F]_{11}$ (▼). ^{19}F NMR spectra (376 MHz, 298K) of (d) the D_2O phase after extraction, showing only the presence of the encapsulated TfO^- without free Tf_2N^-, and (e) the CD_3NO_2 phase after extraction, showing only the presence of free Tf_2N^- without TfO^-. These results indicate that along with the complete removal of TfO^- from the organic phase to the water phase, the exchanged Tf_2N^- transferred from the water phase to the organic phase as the ion pair $TBA^+Tf_2N^-$, exhibiting anion exchange-like behaviour.

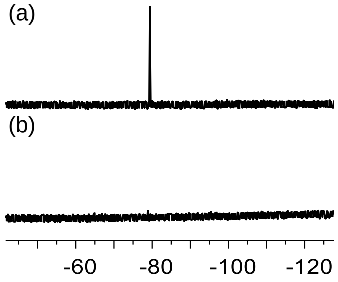

Figure S34. Distribution of TBAOTf between two phases, CD_3NO_2 and D_2O, in the absence of cage extractant in D_2O (conditions: 0.8 mM TBAOTf in 600 µl CD_3NO_2; 600 µl pure D_2O; 3 hours of stirring at rt). ^{19}F NMR spectra (376 MHz, 298K) of (a) the CD_3NO_2 phase and (b) D_2O phase after thoroughly mixing for 3 h.

7. References

[1] J. G. Park, R. Jeon Ie, T. D. Harris, *Inorg. Chem.* **2015**, *54*, 359-369.

[2] D. Zhang, T. K. Ronson, J. Mosquera, A. Martinez, L. Guy, J. R. Nitschke, *J. Am. Chem. Soc.* **2017**, *139*, 6574-6577.

[3] A. Martinez, V. Robert, H. Gornitzka, J.-P. Dutasta, *Chem. Eur. J.* **2010**, *16*, 520-527.

[4] A. M. Castilla, T. K. Ronson, J. R. Nitschke, *J. Am. Chem. Soc.* **2016**, *138*, 2342-2351.

[5] Bruker-Nonius, *APEX, SAINT and XPREP*, Bruker AXS Inc., Madison, Wisconsin, USA, **2013**.

[6] a) N. Collaborative Computational Project, *Acta Cryst.* **1994**, *D50*, 760-763; b) P. Evans, *Acta Cryst.* **2006**, *D62*, 72-82; c) G. Winter, *J. Appl. Crystallogr.* **2010**, *43*, 186-190.

[7] L. Farrugia, *J. Appl. Crystallogr.* **2012**, *45*, 849-854.

[8] P. R. Evans, G. N. Murshudov, *Acta Crystallogr., Sect. D* **2013**, *69*, 1204-1214.

[9] M. D. Winn, C. C. Ballard, K. D. Cowtan, E. J. Dodson, P. Emsley, P. R. Evans, R. M. Keegan, E. B. Krissinel, A. G. W. Leslie, A. McCoy, S. J. McNicholas, G. N. Murshudov, N. S. Pannu, E. A. Potterton, H. R. Powell, R. J. Read, A. Vagin, K. S. Wilson, *Acta Crystallogr., Sect. D* **2011**, *67*, 235-242.

[10] G. Sheldrick, *Acta. Cryst.* **2015**, *A71*, 3-8.

[11] G. M. Sheldrick, *Acta. Cryst.* **2015**, *C71*, 3-8.

[12] P. van der Sluis, A. L. Spek, *Acta Cryst.* **1990**, *A46*, 194-201.

[13] A. L. Spek, *PLATON: A Multipurpose Crystallographic Tool*, Utrecht University, Utrecht, The Netherlands, **2008**.

[14] G. J. Kleywegt, T. A. Jones, *Acta Cryst.* **1994**, *D50*, 178-185.

[15] Y. R. Hristova, M. M. J. Smulders, J. K. Clegg, B. Breiner, J. R. Nitschke, *Chem. Sci.* **2011**, *2*, 638-641.
